# Pd-catalyzed [3 + 2] cycloaddition of vinylcyclopropanes with 1-azadienes: synthesis of 4-cyclopentylbenzo[*e*][1,2,3]oxathiazine 2,2-dioxides†

**DOI:** 10.1039/c8ra08881k

**Published:** 2018-12-05

**Authors:** Yan Lin, Qijun Wang, Yang Wu, Chang Wang, Hao Jia, Cheng Zhang, Jiaxing Huang, Hongchao Guo

**Affiliations:** Department of Applied Chemistry, China Agricultural University Beijing 100193 China hchguo@cau.edu.cn 05084@cau.edu.cn

## Abstract

The palladium-catalyzed [3 + 2] cycloaddition of vinylcyclopropanes and 1-azadienes has been developed under mild reaction conditions, giving the multisubstituted cyclopentane derivatives in good to excellent yields with moderate to good diastereoselectivities. The relative configuration of both diastereomers of the products have been determined through X-ray crystallographic diffraction.

## Abstract 2

Pd-catalyzed [3 + 2] cycloaddition of vinylcyclopropanes with 1-azadienes gave highly functionalized cyclopentane derivatives in high yields.

## Graphical abstract

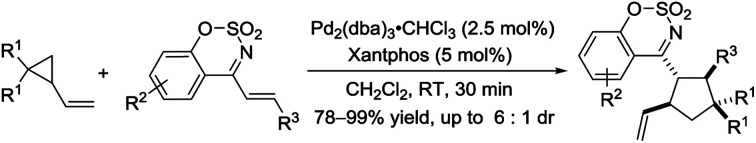

The cyclopentane framework is ubiquitous in nature and it is also an important structural moiety in many pharmaceuticals, agrochemicals, and materials.^1^ The development of simple, fast and efficient synthetic methods for highly substituted cyclopentanes has attracted much attention. Among various methods for synthesis of cyclopentane structure, the cycloaddition reaction is a very attractive one.^2^

Under palladium catalysis conditions, vinylcyclopropane derivatives underwent a ring-opening reaction to generate the zwitterionic allylpalladium intermediate, which reacted with carbon–carbon, carbon–oxygen, carbon–nitrogen double bonds and diazo compounds to provide a variety of five-membered cyclic compounds.^3^ In the past decades, this type of annulation reactions has emerged as a powerful tool for the synthesis of carbocyclic and heterocyclic compounds.^2^ Diverse substrates including isocyanates,^4^ aldehydes,^5^ isatins,^6^ 3-diazooxindoles,^7^ electron-deficient alkenes such as para-quinone methides,^8^ α,β-unsaturated aldehydes,^9^ β,γ-unsaturated α-keto esters,^10^ nitroolefins,^11^ azlactone- and Meldrum's acid alkylidenes,^12^ and α-nucleobase substituted acrylates,^13^ have been exploited in palladium-catalyzed [3 + 2] cycloadditions of vinyl cyclopropane, delivering biologically interesting functionalized heterocyclic compounds and cyclopentane derivatives. In 2015, Liu and He reported a palladium-catalyzed [3 + 2] cycloaddition of vinyl cyclopropane and α,β-unsaturated imines generated *in situ* from aryl sulfonyl indoles, providing the optically enriched spirocyclopentane-1,3′-indolenines with high diastereoselectivity.^14^ This is the only example where α,β-unsaturated imines were employed in Pd-catalyzed [3 + 2] cycloaddition reaction of vinyl cyclopropane.

As a type of α,β-unsaturated imines, cyclic 1-azadienes such as (*E*)-4-styrylbenzo[*e*][1,2,3]oxathiazine 2,2-dioxides 2 are easily accessible and stable (Scheme 1). In particular, they contain the sulfonate-moiety, which is an interesting biologically important motif, and has a great potential in the synthesis of bioactive molecules.^15^ The cyclic 1-azadienes have been used in a series of annulation reactions such as [2 + *n*],^16–18^ [3 + *n*],^19^ and [4 + *n*]^20^ annulation reactions. Based on the electron-deficient nature of the carbon–carbon double bond in these cyclic 1-azadienes, in 2016, Chen and Ouyang developed cinchona-derived tertiary amine-catalyzed asymmetric [3 + 2] annulation of isatin-derived Morita–Baylis–Hillman (MBH) carbonates with cyclic 1-azadienes to form spirooxindole.^17^ In the same year, our group demonstrated a phosphine-catalyzed [3 + 2] annulation of MBH carbonates with cyclic 1-azadienes.^18^ Considering that the 1-azadiene 2 is an electron-deficient alkene with good reactivity, its reaction with a zwitterionic π-allyl Pd complex formed *via* ring-opening of vinylcyclopropane may be feasible. However, this type of cyclic 1-azadienes have never been used in Pd-catalyzed annulation reactions involving vinylcyclopropanes. As our continuing interest on cycloaddition reactions,^21^ herein we disclose a [3 + 2] cycloaddition of palladium-catalyzed vinyl cyclopropane with cyclic 1-azadienes to afford the multisubstituted cyclopentane derivatives (Scheme 1).

**Scheme 1:**
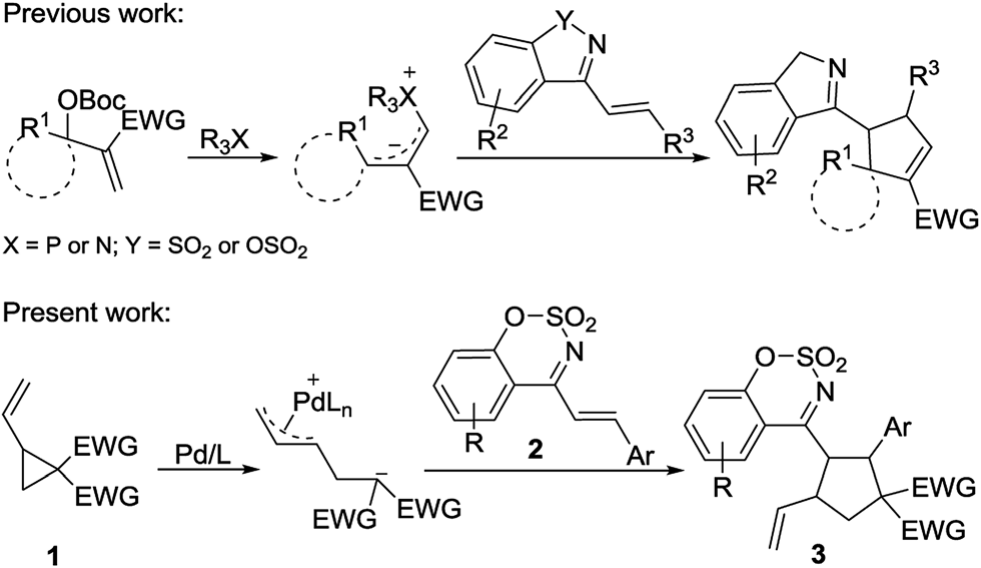
[3 + 2] Cycloaddition of 1,3-zwitterions with cyclic 1-azadienes.

We carried out an initial screening with 2-vinylcyclopropane-1,1-dicarbonitrile 1a and (*E*)-4-styrylbenzo[*e*][1,2,3]oxathiazine 2,2-dioxide 2a in CH_2_Cl_2_ (DCM) at room temperature in the presence of Pd_2_(dba)_3_·CHCl_3_ (2.5 mol%) (Table 1, entry 1). The reaction worked but required 24 hours to make full conversion, furnishing the desired [3 + 2] cycloadduct 3 in 96% yield with 5 : 1 dr (entry 1). Several phosphines including PPh_3_ and bidentate phosphines were next screened as ligands. It was found that the reaction efficiency was remarkably increased in the presence of phosphines (entries 2–6). With the use of the diphoshhine Xantphos as ligand, the reaction time was shortened to 0.5 h and the yield remained at 96%. Decreasing the catalyst loading to 1%, the product could still be obtained in 86%yield with 6 : 1 dr (entry 7). A quick screening of solvents such as toluene, THF, 1,2-dichloroethane (DCE) and MeCN was performed. When toluene and THF were employed as the solvent, the nearly same results as that in CH_2_Cl_2_ were observed (entries 8–9). However, using DCE or MeCN as solvent led to a slightly lower diastereoselectivity (entries 10–11). On the basis of the above investigation, the optimal reaction conditions was determined as follow: using Pd_2_(dba)_3_·CHCl_3_ (2.5 mol%) and Xantphos (5.0 mol%) as catalyst in CH_2_Cl_2_ at room temperature. The relative configurations of two diastereomers were determined by single crystal X-ray analysis of the product 3aa and 3aa′.^22^ Under the optimized reaction conditions, we attempted to develop asymmetric variant of this reaction. Unfortunately, the two enantiomers of the product could not be resolved at present stage.^23^

**Table 1:** Optimization of the reaction conditions^a^

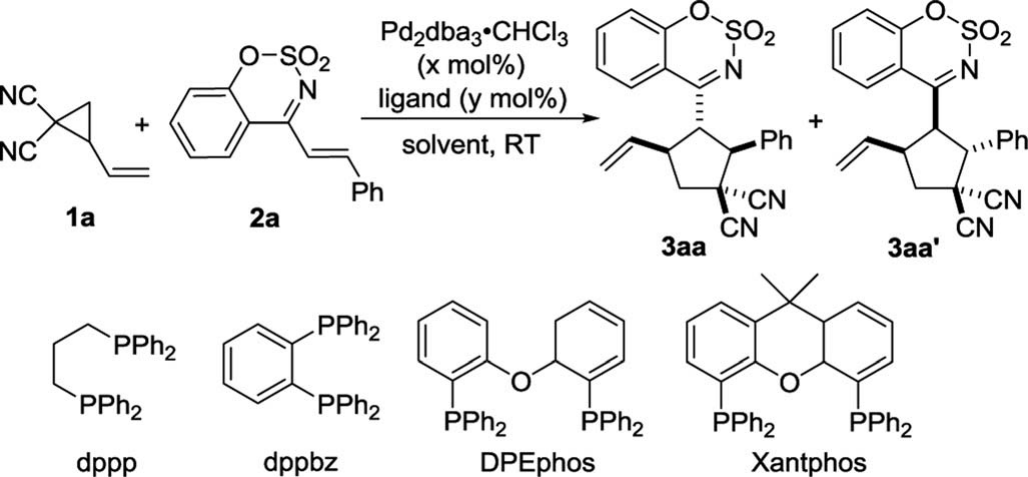						
Entry	*x*	L (*y* mol%)	Solvent	t/h	Yield (%)	3aa : 3aa′^b^
1	2.5	—	DCM	24	96	5 : 1
2	2.5	PPh_3_ (10)	DCM	5	81	3 : 1
3	2.5	dppp (5.0)	DCM	5	97	5 : 1
4	2.5	DPEphos (5)	DCM	5	89	2 : 1
5	2.5	dppbz (5)	DCM	15	95	6 : 1
6	2.5	Xantphos (5)	DCM	0.5	96	6 : 1
7	1.0	Xantphos (2)	DCM	0.5	86	6 : 1
8	2.5	Xantphos (5)	Toluene	0.5	97	6 : 1
9	2.5	Xantphos (5)	THF	0.5	92	6 : 1
10	2.5	Xantphos (5)	DCE	1.5	99	4 : 1
11	2.5	Xantphos (5)	MeCN	5	68	5 : 1

a Unless otherwise stated, all reactions were carried out with 1a (0.12 mmol), 2a (0.10 mmol) and catalyst in solvent (2 mL) at room temperature.

b Determined by isolated yield.

Having the optimized reaction condition in hand, the generality of Pd-catalyzed [3 + 2] cycloaddition of 2-vinylcyclopropane-1,1-dicarbonitrile 1a was scrutinized by using a series of cyclic 1-azadienes 2b–2n (Table 2, entries 2–14). A wide range of substituents on the 1-azadienes 2 were well tolerated in the reaction with the 2-vinylcyclopropane-1,1-dicarbonitrile 1a, giving desired cycloadducts 3aa–3an in high to excellent yields (78–99%) with moderate diastereoselectivities (2 : 1–6 : 1 dr). Regardless of electron-donating groups such as Me and MeO, electron-withdrawing groups such as F, Cl and Br, and their positions on benzene ring, the yields of the corresponding products were satisfactory. Interestingly, 4-Cl, 4-Br or 3,4-dimethoxy substituted 1-azadiene delivered a relative lower yield of product, compared with other substrates (entries 5, 6, 13 *vs.* 1–4, 7–12). The yield significantly decreased to 78% when a electron-donating methyl group was introducted onto benzo[*e*][1,2,3] oxathiazine 2,2-dioxide moiety (entry 14). When employing dimethyl 2-vinylcyclopropane-1,1-dicarboxylate 1b instead of 2-vinylcyclopropane-1,1-dicarbonitrile 1a to react with 2a, the corresponding product 3ba was obtained in 86% yield albeit with a 2 : 1 dr (entry 15).

**Table 2:** The scope of 1-azadienes^a^

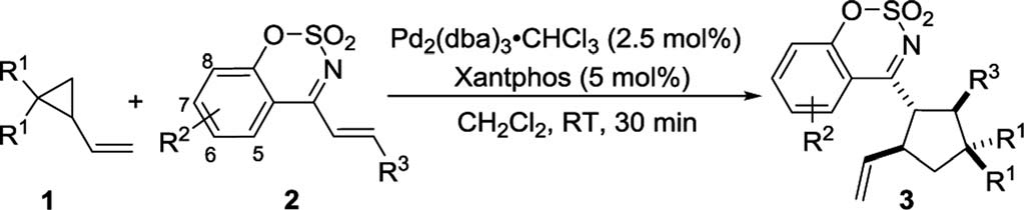					
Entry	R^1^ in 1	R^2^, R^3^ in 2	3	Yield (%)	dr^b^
1	CN (1a)	H, Ph (2a)	3aa	96	6 : 1
2	CN (1a)	H, 2-FC_6_H_4_ (2b)	3ab	98	4 : 1
3	CN (1a)	H, 3-FC_6_H_4_ (2c)	3ac	95	4 : 1
4	CN (1a)	H, 4-FC_6_H_4_ (2d)	3ad	98	2 : 1
5	CN (1a)	H, 4-ClC_6_H_4_ (2e)	3ae	81	3 : 1
6	CN (1a)	H, 4-BrC_6_H_4_ (2f)	3af	80	3 : 1
7	CN (1a)	H, 2-MeC_6_H_4_ (2g)	3ag	99	2 : 1
8	CN (1a)	H, 3-MeC_6_H_4_ (2h)	3ah	95	3 : 1
9	CN (1a)	H, 4-MeC_6_H_4_ (2i)	3ai	95	3 : 1
10	CN (1a)	H, 2-OMeC_6_H_4_ (2j)	3aj	99	2 : 1
11	CN (1a)	H, 3-OMeC_6_H_4_ (2k)	3ak	94	3 : 1
12	CN (1a)	H, 4-OMeC_6_H_4_ (2l)	3al	99	2 : 1
13	CN (1a)	H, 3,4-OMe_2_C_6_H_3_ (2m)	3am	80	2 : 1
14	CN (1a)	6-Me, Ph (2n)	3an	78	2 : 1
15^c^	CO_2_Me (1b)	H, Ph (2a)	3ba	86	2 : 1

a Unless otherwise stated, all reactions were carried out with 1 (0.18 mmol), 2 (0.15 mmol) and catalyst in CH_2_Cl_2_ (3 mL) at room temperature for 30 minutes.

b Determined by ^1^H NMR analysis.

c After 24 h, the starting material was completely consumed (monitored by TLC).

To further demonstrate the reaction to be a practical tool for the synthesis of polysubstituted cyclopentane derivatives, the reaction was carried out on the gram scale. We were satisfied to found that when decreasing the loading of palladium/ligand to 0.5%/1.0%, the reaction still worked very efficiently and completed in one hour to provide the product 3aa in 92% yield with 7 : 1 dr (Scheme 2).

**Scheme 2:**
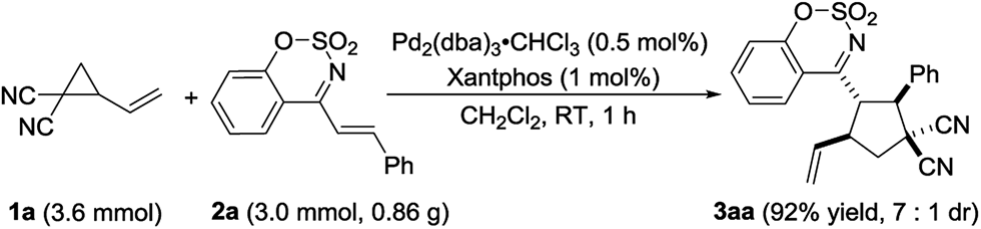
Reaction on the gram scale.

## Conclusions

In conclusion, we have developed a new method to access the functionalized polysubstituted cyclopentane derivatives in good to excellent yields, employing palladium-catalyzed [3 + 2] cycloaddition between vinylcyclopropanes and 1-azadienes under mild reaction conditions. The reaction tolerated a wide range of substrates and could be performed on the gram scale, showing that it is a practical tool for synthesis of biologically interesting cyclopentane derivatives.

## Experimental

### General methods

All reactions were performed under argon atmosphere. Infrared spectra were recorded using an FT-IR spectrophotometer. ^1^H and ^13^C NMR spectra were recorded using a 300 MHz NMR instrument. Accurate mass measurements were performed on an electrospray ionization (ESI) apparatus using time-of-flight (TOF) mass spectrometry. Melting points were determined on a melting apparatus. 1,1-Disubstituted-2-vinylcyclopropanes 1 were prepared according to the literature procedure.^24^ (*E*)-4-Styrylbenzo[*e*][1,2,3]oxathiazine 2,2-dioxides 2 were synthesized according to the literature procedure.^25^

### General procedure for [3 + 2] annulation reaction

An oven-dried 10 mL of Schlenk tube was charged with 1-azadiene 2 (0.15 mmol), vinylcyclopropane 1 (1.2 equiv., 0.18 mmol), Pd_2_(dba)_3_·CHCl_3_ (0.025 equiv., 3.9 mg), Xantphos (0.05 equiv., 4.3 mg) in 3 mL of CH_2_Cl_2_ for corresponding time under argon atmosphere at room temperature. Once the starting material was completely consumed (monitored by TLC), the mixture was concentrated to dryness. The residue was purified by flash column chromatography (ethyl acetate/petroleum ether = 1/5) to afford the corresponding cycloaddition product 3.

### General procedure for preparation of 3aa on the gram scale

Under argon atmosphere, to a mixture of 1-azadiene 2a (3 mmol, 0.86 g), Pd_2_(dba)_3_·CHCl_3_ (0.005 equiv., 15.5 mg), Xantphos (0.01 equiv., 17.4 mg) in 45 mL of acetonitrile, 2-vinylcyclopropane-1,1-dicarbonitrile 1a (1.2 equiv., 3.6 mmol, 0.42 g) were added at room temperature. The resulting mixture was stirred until the starting material was completely consumed (monitored by TLC) and then was concentrated to dryness. The residue was purified through flash column chromatography (EtOAc/PE 1 : 5) to afford the corresponding annulation product 3aa.

#### 3-(2,2-Dioxidobenzo[*e*][1,2,3]oxathiazin-4-yl)-2-phenyl-4-vinylcyclo-pentane-1,1-dicarbonitrile (3aa)

It has been isolated as a mixture of diastereoisomers. Orange solid (58 mg, 96% yield): mp 198–200 °C; ^1^H NMR (300 MHz, CDCl_3_) *δ* 7.85–7.69 (m, 2H), 7.56–7.47 (m, 2H), 7.47–7.35 (m, 4H), 7.29 (dd, *J* = 8.3, 0.9 Hz, 1H), 5.60 (dt, *J* = 16.8, 9.7 Hz, 1H), 5.09–4.95 (m, 2H), 4.62–4.57 (m, 2H), 3.76 (tdt, *J* = 9.9, 7.0, 5.2 Hz, 1H), 3.02 (dd, *J* = 13.6, 7.2 Hz, 1H), 2.62 (dd, *J* = 13.6, 10.0 Hz, 1H); ^13^C NMR (75 MHz, DMSO-*d*_6_) *δ* 178.6, 152.6, 138.5, 135.0, 133.7, 130.4, 129.1, 129.0, 128.3, 126.8, 118.7, 116.3, 115.6, 114.8, 54.2, 48.6, 44.6, 42.0, 41.4; IR (film) *v*_max_ 1595, 1554, 1448, 1390, 1277, 1267, 1188, 934, 864, 847, 757, 737, 700, 577 cm^−1^; HRMS (ESI) calcd for C_22_H_16_N_3_O_3_S^−^ [M − H]^−^ 402.0918, found 402.0915.

#### 3-(2,2-Dioxidobenzo[*e*][1,2,3]oxathiazin-4-yl)-2-(2-fluorophenyl)-4-vinylcyclopentane-1,1-dicarbonitrile (3ab)

It has been isolated as a mixture of diastereoisomers. White solid (62 mg, 98% yield): mp 156–158 °C; ^1^H NMR (300 MHz, DMSO-*d*_6_) *δ* 8.50 (dd, *J* = 8.1, 1.5 Hz, 1H), 7.94–7.86 (m, 1H), 7.77 (td, *J* = 7.8, 1.4 Hz, 1H), 7.59–7.42 (m, 3H), 7.28 (dtd, *J* = 15.2, 8.2, 1.3 Hz, 2H), 5.71–5.57 (m, 1H), 5.13 (dd, *J* = 11.7, 9.8 Hz, 1H), 4.94 (d, *J* = 3.2 Hz, 1H), 4.87–4.75 (m, 2H), 3.83–3.68 (m, 1H), 3.17 (dd, *J* = 13.9, 7.7 Hz, 1H), 2.86 (dd, *J* = 13.8, 7.4 Hz, 1H); ^13^C NMR (75 MHz, DMSO-*d*_6_) *δ* 178.4, 162.5, 159.2, 152.7, 138.5, 134.6, 131.1 (d, *J* = 8.5 Hz), 130.3, 129.0 (d, *J* = 2.9 Hz), 126.8, 125.0 (d, *J* = 3.3 Hz), 120.9 (d, *J* = 13.8 Hz), 118.8 (d, *J* = 11.6 Hz), 116.1, 115.9 (d, *J* = 22.3 Hz), 115.4, 114.8, 50.3, 49.1, 46.9, 44.9, 42.1; IR (film) *v*_max_ 1595, 1558, 1456, 1447, 1408, 1394, 1275, 1261, 1189, 869, 852, 764, 752 cm^−1^; HRMS (ESI) calcd for C_22_H_15_FN_3_O_3_S^−^ [M − H]^−^ 420.0824, found 420.0824.

#### 3-(2,2-Dioxidobenzo[*e*][1,2,3]oxathiazin-4-yl)-2-(3-fluorophenyl)-4-vinylcyclopentane-1,1-dicarbonitrile (3ac)

It has been isolated as a mixture of diastereoisomers. White solid (60 mg, 95% yield): mp 201–204 °C; ^1^H NMR (300 MHz, DMSO-*d*_6_) *δ* 8.50 (dd, *J* = 8.1, 1.5 Hz, 1H), 7.88 (ddd, *J* = 8.7, 7.5, 1.5 Hz, 1H), 7.58–7.38 (m, 5H), 7.28–7.20 (m, 1H), 5.68–5.49 (m, 1H), 5.08 (dd, *J* = 11.8, 10.2 Hz, 1H), 4.83–4.66 (m, 3H), 3.74 (p, *J* = 8.1 Hz, 1H), 3.16 (dd, *J* = 13.8, 7.8 Hz, 1H), 2.70 (dd, *J* = 13.8, 7.9 Hz, 1H); ^13^C NMR (75 MHz, DMSO-*d*_6_) *δ* 178.5, 163.9, 160.7, 152.7, 138.5, 136.6 (d, *J* = 7.5 Hz), 134.9, 131.1 (d, *J* = 8.5 Hz), 130.5, 126.7, 125.0 (d, *J* = 2.5 Hz), 118.7 (d, *J* = 6.8 Hz), 116.2, 115.3 (d, *J* = 14.1 Hz), 114.8 (d, *J* = 16.8 Hz), 53.5, 48.7, 44.6, 41.9, 41.2; IR (film) *v*_max_ 1593, 1558, 1436, 1388, 1275, 1260, 1188, 852, 786, 764, 750, 703, 553 cm^−1^; HRMS (ESI) calcd for C_22_H_15_FN_3_O_3_S^−^ [M − H]^−^ 420.0824, found 420.0824.

#### 3-(2,2-Dioxidobenzo[*e*][1,2,3]oxathiazin-4-yl)-2-(4-fluorophenyl)-4-vinylcyclopentane-1,1-dicarbonitrile (3ad)

It has been isolated as a mixture of diastereoisomers. White solid (62 mg, 98% yield): mp 207–210 °C; ^1^H NMR (300 MHz, DMSO-*d*_6_) *δ* 8.52 (dd, *J* = 8.2, 1.5 Hz, 1H), 7.89 (ddd, *J* = 8.7, 7.4, 1.4 Hz, 1H), 7.69–7.60 (m, 3H), 7.49 (dd, *J* = 8.3, 1.0 Hz, 1H), 7.34–7.25 (m, 2H), 5.63 (dt, *J* = 17.3, 9.6 Hz, 1H), 5.12–5.00 (m, 1H), 4.86–4.76 (m, 2H), 4.69 (d, *J* = 11.8 Hz, 1H), 3.77 (p, *J* = 8.7 Hz, 1H), 3.16 (dd, *J* = 13.7, 7.9 Hz, 1H), 2.72 (dd, *J* = 13.8, 7.9 Hz, 1H); ^13^C NMR (75 MHz, DMSO-*d*_6_) *δ* 178.5, 164.1, 160.8, 152.6, 138.5, 135.0, 130.8 (d, *J* = 8.5 Hz), 130.6, 130.4 (d, *J* = 2.6 Hz), 126.8, 118.7, 116.2 (d, *J* = 5.6 Hz), 116.0 (d, *J* = 21.5 Hz), 115.5, 53.4, 48.8, 44.6, 41.8, 41.4; IR (film) *v*_max_ 1595, 1558, 1514, 1394, 1275, 1263, 1189, 868, 853, 764, 750, 703, 579, 561, 511 cm^−1^; HRMS (ESI) calcd for C_22_H_15_FN_3_O_3_S^−^ [M − H]^−^ 420.0824, found 420.0825.

#### 2-(4-Chlorophenyl)-3-(2,2-dioxidobenzo[*e*][1,2,3]oxathiazin-4-yl)-4-vinylcyclopentane-1,1-dicarbonitrile (3ae)

It has been isolated as a mixture of diastereoisomers. Yellow solid (53 mg, 81% yield): mp 205–207 °C; ^1^H NMR (300 MHz, DMSO-*d*_6_) *δ* 8.49 (dd, *J* = 8.1, 1.5 Hz, 1H), 7.93–7.83 (m, 1H), 7.64–7.55 (m, 3H), 7.52 (s, 1H), 7.51–7.45 (m, 2H), 5.71–5.53 (m, 1H), 5.09–4.98 (m, 1H), 4.82–4.72 (m, 2H), 4.67 (d, *J* = 11.8 Hz, 1H), 3.75 (ddd, *J* = 17.7, 9.8, 7.7 Hz, 1H), 3.15 (dd, *J* = 13.8, 7.8 Hz, 1H), 2.70 (dd, *J* = 13.9, 7.8 Hz, 1H); ^13^C NMR (75 MHz, DMSO-*d*_6_) *δ* 178.5, 152.6, 138.5, 135.0, 133.9, 132.8, 130.4, 130.2, 129.1, 126.8, 118.7, 116.2, 115.4, 114.7, 53.3, 48.7, 44.5, 41.8, 41.2; IR (film) *v*_max_ 1595, 1553, 1496, 1393, 1275, 1267, 1189, 1094, 866, 850, 790, 764, 751 cm^−1^; HRMS (ESI) calcd for C_22_H_15_ClN_3_O_3_S^−^ [M − H]^−^ 436.0528, found 436.0528.

#### 2-(4-Bromophenyl)-3-(2,2-dioxidobenzo[*e*][1,2,3]oxathiazin-4-yl)-4-vinylcyclopentane-1,1-dicarbonitrile (3af)

It has been isolated as a mixture of diastereoisomers. White solid (58 mg, 80% yield): mp 195–197 °C; ^1^H NMR (300 MHz, DMSO-*d*_6_) *δ* 8.49 (dd, *J* = 8.1, 1.5 Hz, 1H), 7.87 (ddd, *J* = 8.6, 7.5, 1.5 Hz, 1H), 7.64 (d, *J* = 8.5 Hz, 2H), 7.54 (d, *J* = 8.6 Hz, 3H), 7.47 (dd, *J* = 8.3, 1.0 Hz, 1H), 5.72–5.48 (m, 1H), 5.06–4.96 (m, 1H), 4.84–4.71 (m, 2H), 4.66 (d, *J* = 11.9 Hz, 1H), 3.75 (p, *J* = 8.0 Hz, 1H), 3.14 (dd, *J* = 13.8, 7.8 Hz, 1H), 2.70 (dd, *J* = 13.8, 7.8 Hz, 1H); ^13^C NMR (75 MHz, DMSO-*d*_6_) *δ* 178.4, 152.6, 138.5, 135.0, 133.2, 132.0, 130.7, 130.5, 126.8, 122.5, 118.7, 116.2, 115.4, 114.7, 53.4, 48.7, 44.6, 41.8, 41.1; IR (film) *v*_max_ 1595, 1554, 1491, 1393, 1275, 1267, 1189, 1011, 865, 849, 790, 764, 751 cm^−1^; HRMS (ESI) calcd for C_22_H_15_BrN_3_O_3_S^−^ [M − H]^−^ 482.0004, found 482.0007.

#### 3-(2,2-Dioxidobenzo[*e*][1,2,3]oxathiazin-4-yl)-2-(*o*-tolyl)-4-vinylcy-cyclopentane-1,1-dicarbonitrile (3ag)

It has been isolated as a mixture of diastereoisomers. Yellow solid (62 mg, 99% yield): mp 197–198 °C; ^1^H NMR (300 MHz, DMSO-*d*_6_) *δ* 8.48 (dd, *J* = 8.1, 1.5 Hz, 1H), 7.87 (ddd, *J* = 8.5, 7.5, 1.4 Hz, 1H), 7.78–7.72 (m, 1H), 7.60–7.52 (m, 1H), 7.47 (dd, *J* = 8.3, 1.1 Hz, 1H), 7.29–7.20 (m, 3H), 5.65 (dt, *J* = 16.9, 9.7 Hz, 1H), 5.09–4.95 (m, 2H), 4.91–4.74 (m, 2H), 3.82 (p, *J* = 8.7 Hz, 1H), 3.15 (dd, *J* = 13.7, 7.5 Hz, 1H), 2.90 (dd, *J* = 13.6, 8.1 Hz, 1H), 2.60 (s, 3H); ^13^C NMR (75 MHz, DMSO-*d*_6_) *δ* 178.8, 152.6, 138.5, 137.9, 134.8, 132.2, 130.9, 130.4, 128.7, 126.9, 126.8, 126.4, 118.7, 116.2, 115.8, 115.0, 51.0, 49.5, 44.9, 42.5, 40.2, 19.6; IR (film) *v*_max_ 1594, 1558, 1507, 1457, 1448, 1393, 1275, 1263, 1189, 864, 764, 749, 703, 669 cm^−1^; HRMS (ESI) calcd for C_23_H_18_N_3_O_3_S^−^ [M − H]^−^ 416.1074, found 416.1079.

#### 3-(2,2-Dioxidobenzo[*e*][1,2,3]oxathiazin-4-yl)-2-(*m*-tolyl)-4-vinylcy-cyclopentane-1,1-dicarbonitrile (3ah)

It has been isolated as a mixture of diastereoisomers. Yellow solid (60 mg, 95% yield): mp 217–219 °C; ^1^H NMR (300 MHz, DMSO-*d*_6_) *δ* 8.52 (dd, *J* = 8.2, 1.5 Hz, 1H), 7.89 (ddd, *J* = 8.5, 7.5, 1.5 Hz, 1H), 7.61–7.56 (m, 1H), 7.51–7.47 (m, 1H), 7.40 (s, 1H), 7.34–7.19 (m, 3H), 5.61 (dt, *J* = 16.8, 9.8 Hz, 1H), 5.10–4.95 (m, 1H), 4.88–4.71 (m, 2H), 4.58 (d, *J* = 11.7 Hz, 1H), 3.77 (p, *J* = 8.6 Hz, 1H), 3.14 (dd, *J* = 13.7, 7.6 Hz, 1H), 2.73 (dd, *J* = 13.8, 8.2 Hz, 1H), 2.31 (s, 3H); ^13^C NMR (75 MHz, DMSO-*d*_6_) *δ* 178.8, 152.6, 138.5, 138.2, 134.9, 133.7, 130.4, 129.8, 128.9, 128.8, 126.8, 125.4, 118.71, 116.3, 115.6, 114.8, 54.2, 48.6, 44.7, 42.0, 41.5, 21.2; IR (film) *v*_max_ 1594, 1552, 1452, 1394, 1274, 1263, 1189, 856, 790, 764, 749, 703, 558 cm^−1^; HRMS (ESI) calcd for C_23_H_18_N_3_O_3_S^−^ [M − H]^−^ 416.1074, found 416.1075.

#### 3-(2,2-Dioxidobenzo[*e*][1,2,3]oxathiazin-4-yl)-2-(*p*-tolyl)-4-vinylcy-cyclopentane-1,1-dicarbonitrile (3ai)

It has been isolated as a mixture of diastereoisomers. White solid (59 mg, 95% yield): mp 188–191 °C; ^1^H NMR (300 MHz, DMSO-*d*_6_) *δ* 8.52 (dd, *J* = 8.1, 1.5 Hz, 1H), 7.89 (ddd, *J* = 8.5, 7.5, 1.5 Hz, 1H), 7.63–7.55 (m, 1H), 7.51–7.42 (m, 3H), 7.24 (d, *J* = 8.0 Hz, 2H), 5.62 (dt, *J* = 16.8, 9.8 Hz, 1H), 5.06–4.98 (m, 1H), 4.87–4.78 (m, 2H), 4.60 (d, *J* = 11.8 Hz, 1H), 3.77 (p, *J* = 8.4 Hz, 1H), 3.14 (dd, *J* = 13.8, 7.7 Hz, 1H), 2.73 (dd, *J* = 13.9, 8.1 Hz, 1H), 2.29 (s, 3H); ^13^C NMR (75 MHz, DMSO-*d*_6_) *δ* 178.7, 152.6, 138.6, 138.5, 135.0, 130.6, 130.3, 129.6, 128.1, 126.8, 118.7, 116.3, 115.6, 114.8, 54.0, 48.6, 44.6, 41.9, 41.5, 20.8; IR (film) *v*_max_ 1594, 1553, 1391, 1275, 1262, 1187, 866, 851, 751, 703, 576, 556, 508 cm^−1^; HRMS (ESI) calcd for C_23_H_18_N_3_O_3_S^−^ [M − H]^−^ 416.1074, found 416.1075.

#### 3-(2,2-Dioxidobenzo[*e*][1,2,3]oxathiazin-4-yl)-2-(2-methoxyphenyl)-4-vinylcyclopentane-1,1-dicarbonitrile (3aj)

It has been isolated as a mixture of diastereoisomers. Yellow solid (64 mg, 99% yield): mp 145–148 °C; ^1^H NMR (300 MHz, DMSO-*d*_6_) *δ* 8.56 (dd, *J* = 8.1, 1.5 Hz, 1H), 7.91 (m, 1H), 7.63–7.49 (m, 2H), 7.38 (m, 2H), 7.11 (td, *J* = 8.3, 1.0 Hz, 1H), 6.96 (td, *J* = 7.6, 1.1 Hz, 1H), 5.67 (ddd, *J* = 16.8, 10.1, 9.2 Hz, 1H), 5.17 (dd, *J* = 11.5, 8.5 Hz, 1H), 4.96–4.85 (m, 3H), 3.86 (s, 3H), 3.63 (p, *J* = 7.8 Hz, 1H), 3.14 (dd, *J* = 13.9, 7.1 Hz, 1H), 2.83 (dd, *J* = 13.8, 5.5 Hz, 1H); ^13^C NMR (75 MHz, DMSO-*d*_6_) *δ* 178.8, 157.7, 152.7, 138.6, 134.0, 130.1, 130.0, 127.5, 126.9, 122.6, 120.7, 119.1, 118.9, 116.1, 115.2, 111.7, 59.8, 55.6, 49.0, 48.0, 46.2, 43.0; IR (film) *v*_max_ 1594, 1552, 1495, 1464, 1390, 1294, 1275, 1252, 1188, 1054, 1027, 853, 753, 557, 509 cm^−1^; HRMS (ESI) calcd for C_23_H_18_N_3_O_4_S^−^ [M − H]^−^ 432.1024, found 432.1027.

#### 3-(2,2-Dioxidobenzo[*e*][1,2,3]oxathiazin-4-yl)-2-(3-methoxyphenyl)-4-vinylcyclopentane-1,1-dicarbonitrile (3ak)

It has been isolated as a mixture of diastereoisomers. White solid (61 mg, 94% yield): mp 210–212 °C; ^1^H NMR (300 MHz, DMSO-*d*_6_) *δ* 8.52 (dd, *J* = 8.2, 1.5 Hz, 1H), 7.96–7.84 (m, 1H), 7.58–7.45 (m, 2H), 7.39–7.29 (m, 1H), 7.17–7.07 (m, 2H), 6.96 (dd, *J* = 8.2, 2.0 Hz, 1H), 5.59 (dt, *J* = 17.4, 9.6 Hz, 1H), 5.08–4.89 (m, 2H), 4.85–4.72 (m, 2H), 4.60 (d, *J* = 11.8 Hz, 1H), 3.74 (s, 3H), 3.12 (dd, *J* = 13.8, 7.6 Hz, 1H), 2.71 (dd, *J* = 13.8, 8.2 Hz, 1H); ^13^C NMR (75 MHz, DMSO-*d*_6_) *δ* 178.7, 159.5, 152.6, 138.5, 135.3, 135.0, 130.4, 130.2, 126.8, 120.5, 118.7, 116.3, 115.6, 114.8, 114.4, 114.0, 55.2, 54.1, 48.6, 44.6, 42.0, 41.3; IR (film) *v*_max_ 1594, 1558, 1469, 1455, 1388, 1290, 1275, 1262, 1187, 858, 764, 750, 703, 680, 554 cm^−1^; HRMS (ESI) calcd for C_23_H_18_N_3_O_4_S^−^ [M − H]^−^ 432.1024, found 432.1024.

#### 3-(2,2-Dioxidobenzo[*e*][1,2,3]oxathiazin-4-yl)-2-(4-methoxyphenyl)-4-vinylcyclopentane-1,1-dicarbonitrile (3al)

It has been isolated as a mixture of diastereoisomers. Yellow solid (64 mg, 99% yield): mp 157–160 °C; ^1^H NMR (300 MHz, CDCl_3_) *δ* 7.81–7.72 (m, 2H), 7.44–7.38 (m, 3H), 7.36–7.31 (m, 1H), 6.95–6.87 (m, 2H), 5.57 (dt, *J* = 16.8, 9.7 Hz, 1H), 5.05–4.90 (m, 2H), 4.62–4.47 (m, 2H), 3.77 (s, 3H), 3.76–3.64 (m, 1H), 2.98 (dd, *J* = 13.6, 7.3 Hz, 1H), 2.56 (dd, *J* = 13.6, 9.9 Hz, 1H); ^13^C NMR (75 MHz, CDCl_3_) *δ* 176.6, 160.2, 153.4, 137.3, 132.9, 128.9, 127.6, 125.9, 123.8, 119.8, 119.0, 116.4, 114.4, 114.1, 55.3, 55.0, 48.6, 45.0, 43.5, 41.5; IR (film) *v*_max_ 1609, 1594, 1552, 1516, 1477, 1452, 1392, 1292, 1257, 1189, 1035, 853, 765, 752, 703 cm^−1^; HRMS (ESI) calcd for C_23_H_18_N_3_O_4_S^−^ [M − H]^−^ 432.1024, found 432.1026.

#### 2-(3,4-Dimethoxyphenyl)-3-(2,2-dioxidobenzo[*e*][1,2,3]oxathiazin-4-yl)-4-vinylcyclopentane-1,1-dicarbonitrile (3am)

It has been isolated as a mixture of diastereoisomers. Orange solid (56 mg, 80% yield): mp 99–102 °C; ^1^H NMR (300 MHz, CDCl_3_) *δ* 7.79–7.72 (m, 2H), 7.49–7.40 (m, 1H), 7.28 (t, *J* = 1.1 Hz, 2H), 7.05–7.00 (m, 1H), 6.94–6.82 (m, 2H), 5.57 (dt, *J* = 16.8, 9.7 Hz, 1H), 5.13 (d, *J* = 9.9 Hz, 1H), 5.04–4.93 (m, 1H), 4.57–4.43 (m, 2H), 3.89 (s, 3H), 3.85 (s, 3H), 3.80–3.61 (m, 1H), 2.98 (dd, *J* = 13.6, 7.2 Hz, 1H), 2.56 (dd, *J* = 13.6, 10.0 Hz, 1H); ^13^C NMR (75 MHz, CDCl_3_) *δ* 178.0, 176.5, 153.4, 149.7, 149.1, 137.6, 137.3, 135.7, 132.8, 127.9, 127.5, 125.8, 119.7, 119.1, 116.4, 114.2, 111.3, 55.8, 55.5, 51.7, 48.7, 45.0, 43.5, 41.3; IR (film) *v*_max_ 1593, 1552, 1519, 1465, 1448, 1389, 1260, 1187, 1166, 1147, 1024, 856, 765, 735, 703, 564 cm^−1^; HRMS (ESI) calcd for C_24_H_20_N_3_O_5_S^−^ [M − H]^−^ 462.1129, found 462.1130.

#### 3-(6-Methyl-2,2-dioxidobenzo[*e*][1,2,3]oxathiazin-4-yl)-2-phenyl-4-vinylcyclopentane-1,1-dicarbonitrile (3an)

It has been isolated as a mixture of diastereoisomers. Yellow solid (49 mg, 78% yield): mp 224–227 °C; ^1^H NMR (300 MHz, DMSO-*d*_6_) *δ* 8.33 (s, 1H), 7.67 (dd, *J* = 8.6, 2.0 Hz, 1H), 7.54 (td, *J* = 7.9, 1.6 Hz, 2H), 7.45–7.33 (m, 4H), 5.60 (dt, *J* = 16.9, 9.8 Hz, 1H), 5.09–4.96 (m, 1H), 4.88–4.72 (m, 2H), 4.62 (d, *J* = 11.8 Hz, 1H), 3.77 (p, *J* = 8.7 Hz, 1H), 3.14 (dd, *J* = 13.6, 8.0 Hz, 1H), 2.73 (dd, *J* = 13.8, 8.2 Hz, 1H), 2.45 (s, 3H); ^13^C NMR (75 MHz, DMSO-*d*_6_) *δ* 178.6, 150.7, 139.0, 136.8, 135.1, 133.8, 129.9, 129.0, 128.6, 128.3, 118.6, 118.4, 116.1, 114.9, 54.2, 48.5, 44.6, 41.9, 41.4, 20.3; IR (film) *v*_max_ 1559, 1541, 1467, 1452, 1387, 1275, 1261, 1186, 1143, 832, 764, 750, 698, 557 cm^−1^; HRMS (ESI) calcd for C_23_H_18_N_3_O_3_S^−^ [M − H]^−^ 416.1074, found 416.1075.

#### 3-(2,2-Dioxidobenzo[*e*][1,2,3]oxathiazin-4-yl)-2-phenyl-4-vinylcyclo-pentane-1,1-dicarboxylate (3ba)

It has been isolated as a mixture of diastereoisomers. Orange solid (60 mg, 86% yield): mp 145–149 °C; ^1^H NMR (300 MHz, CDCl_3_) *δ* 7.69–7.63 (m, 1H), 7.61 (s, 1H), 7.33–7.28 (m, 1H), 7.24–7.17 (m, 6H), 5.84 (ddd, *J* = 17.0, 10.2, 8.0 Hz, 1H), 5.09 (dt, *J* = 17.1, 1.1 Hz, 1H), 4.97 (ddd, *J* = 10.2, 1.3, 0.8 Hz, 1H), 4.91–4.81 (m, 1H), 4.64 (d, *J* = 10.7 Hz, 1H), 4.05 (t, *J* = 10.5 Hz, 1H), 3.79 (s, 3H), 3.19 (s, 3H), 2.90 (dd, *J* = 14.0, 11.5 Hz, 1H), 2.58 (dd, *J* = 14.0, 7.8 Hz, 1H); ^13^C NMR (75 MHz, CDCl_3_) *δ* 180.1, 171.2, 153.2, 136.8, 136.63, 136.59, 136.5, 135.7, 128.2, 128.0, 127.8, 127.4, 125.2, 118.7, 117.2, 116.7, 63.9, 55.7, 53.7, 52.7, 52.0, 48.4, 39.5; IR (film) *v*_max_ 1728, 1593, 1551, 1448, 1436, 1391, 1266, 1189, 1088, 926, 857, 752, 701, 566 cm^−1^; HRMS (ESI) calcd for C_24_H_22_NO_7_S^−^ [M − H]^−^ 468.1122, found 468.1124.

## Conflicts of interest

There are no conflicts to declare.

## Supplementary Material

RA-008-C8RA08881K-s001

RA-008-C8RA08881K-s002
