# Supplementary material for: Pd-catalyzed [3 + 2] cycloaddition of vinylcyclopropanes with 1-azadienes: synthesis of 4-cyclopentylbenzo[e][1,2,3]oxathiazine 2,2-dioxides
Source: RSC Adv. 2018 Dec 5;8(71):40798–803. doi: 10.1039/c8ra08881k (PMC9091363; doi:10.1039/c8ra08881k)
Supplement: RA-008-C8RA08881K-s001 [file RA-008-C8RA08881K-s001.pdf]

Electronic Supplementary Material (ESI) for RSC Advances.
This journal is © The Royal Society of Chemistry 2018

Electronic Supplementary Information

Pd-Catalyzed [3+2] Cycloaddition of Vinylcyclopropanes with 1-Azadienes: Synthesis of 4-Cyclopentylbenzo[*e*][1,2,3]oxathiazine 2,2-Dioxides

Yan Lin, Qijun Wang, Yang Wu, Chang Wang, Hao Jia, Cheng Zhang, Jiaxing Huang,* and Hongchao Guo*

Department of Applied Chemistry, China Agricultural University, Beijing 100193, P. R. China

Email: hchguo@cau.edu.cn, 05084@cau.edu.cn

Contents

Copies of ^{1}H and ^{13}C NMR Spectra S01–S17

X-Ray Crystallographic Data of **3aa** and **3aa’** S19–S38

Copies of ^{1}H and ^{13}C NMR Spectra

2-vinylcyclopropane-1,1-dicarbonitrile (1a)

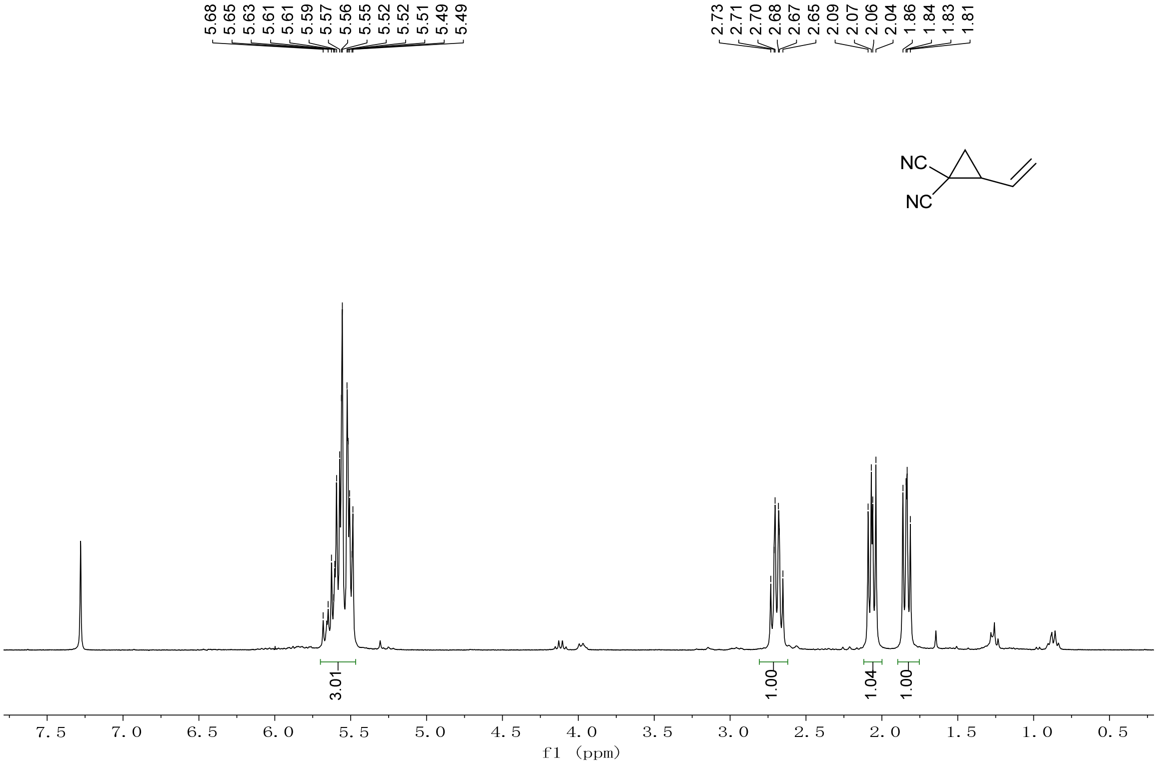

(*E*)-4-(2-fluorostyryl)benzo[*e*][1,2,3]oxathiazine 2,2-dioxide (2b)

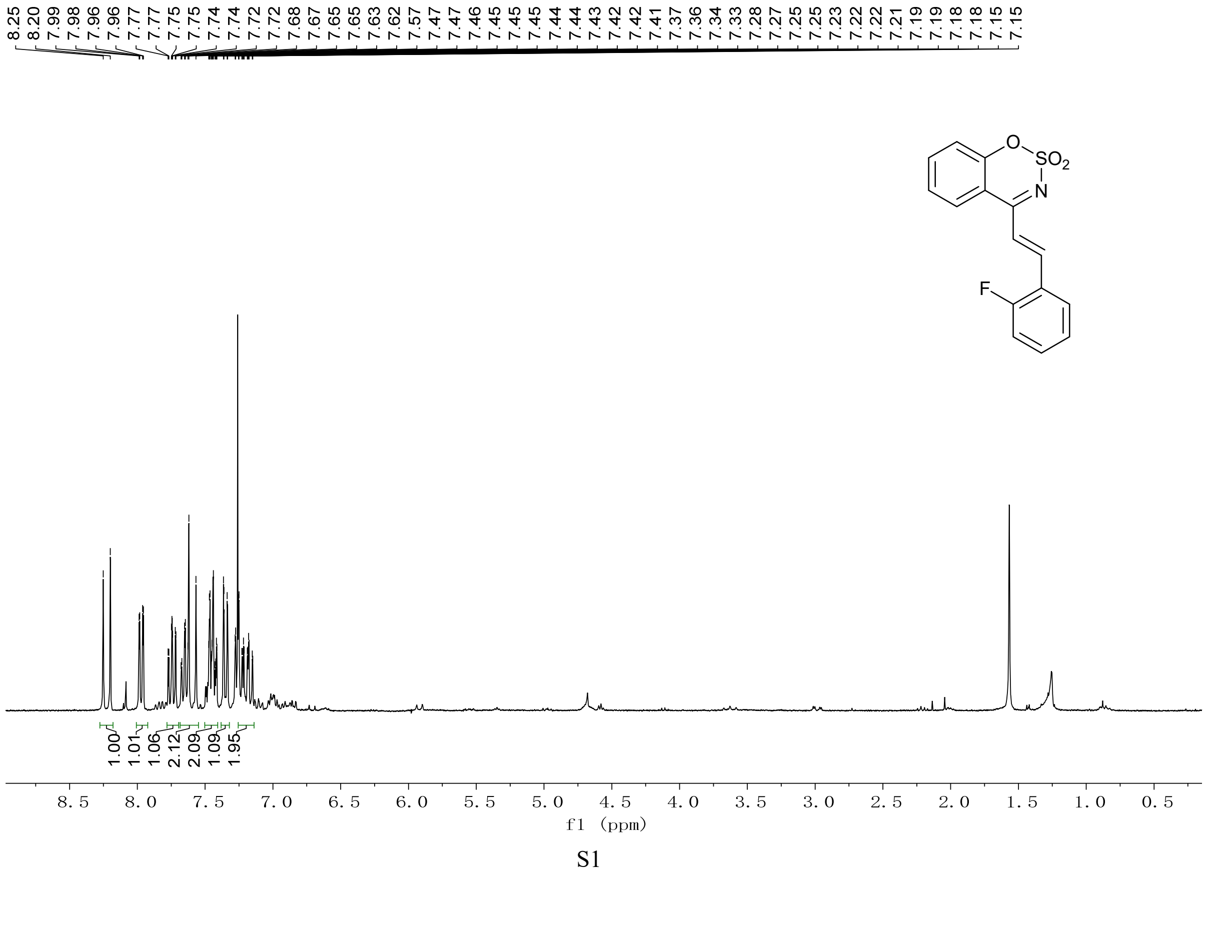

(*E*)-4-(2-methoxystyryl)benzo[*e*][1,2,3]oxathiazine 2,2-dioxide (2j)

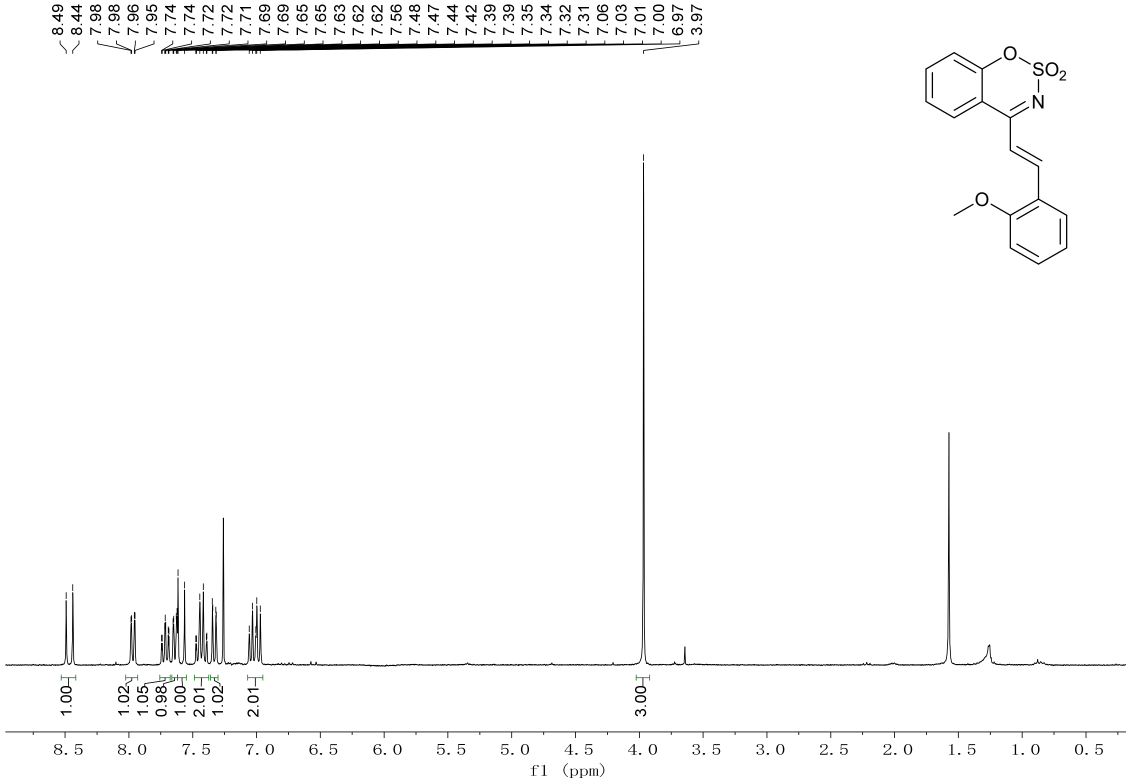

(*E*)-6-methyl-4-styrylbenzo[*e*][1,2,3]oxathiazine 2,2-dioxide (2n)

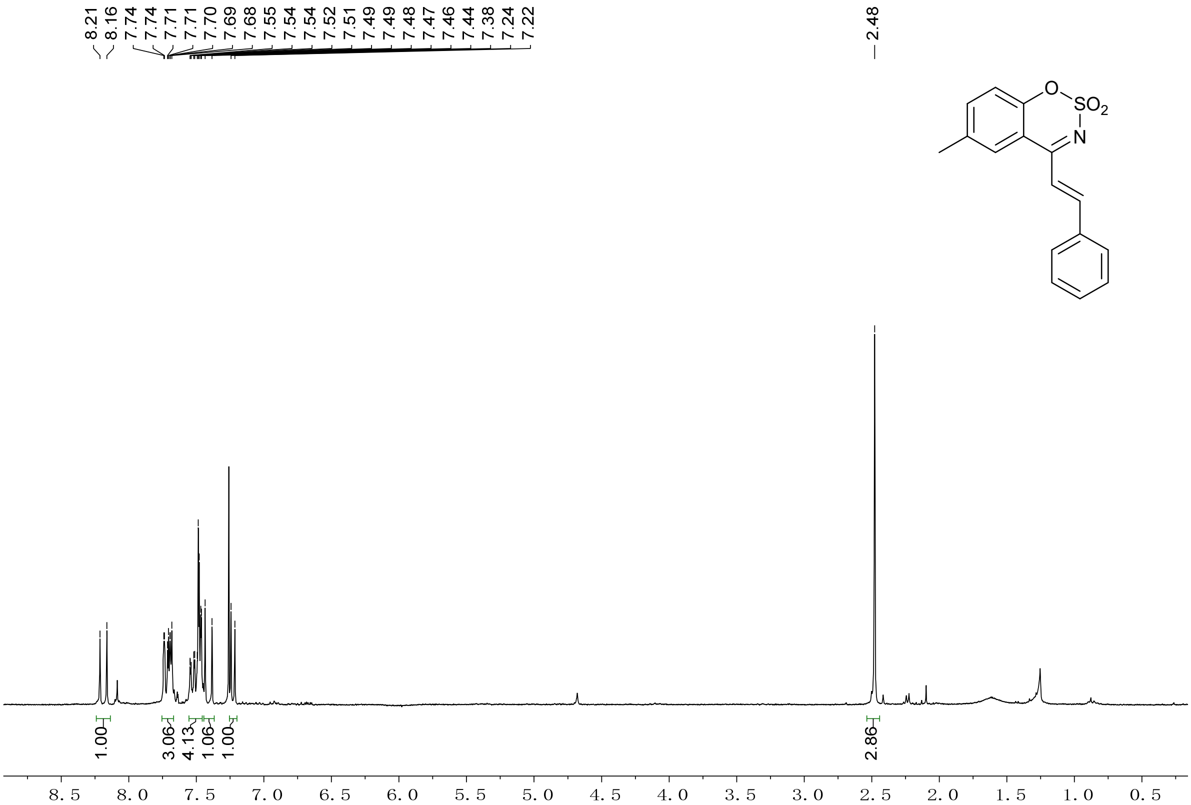

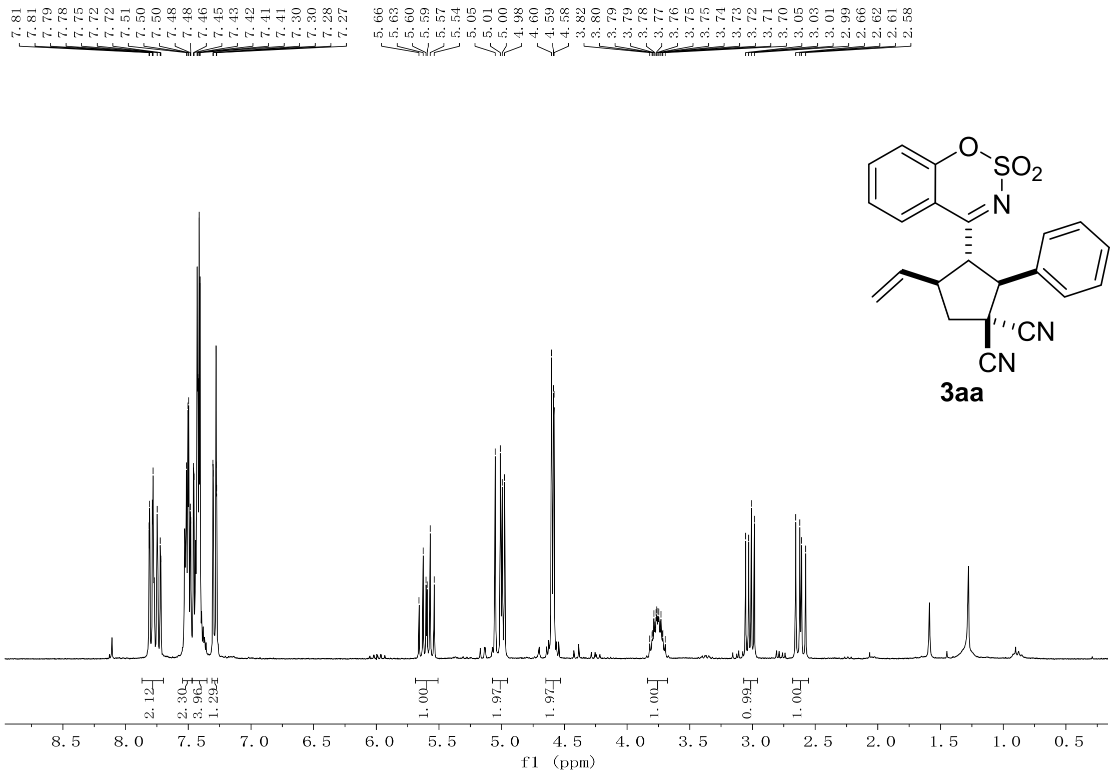
7.81 7.81 7.79 7.78 7.75 7.72 7.72 7.51 7.50 7.50 7.48 7.48 7.46 7.45 7.43 7.42 7.41 7.41 7.30 7.30 7.28 7.27
5.66 5.63 5.60 5.59 5.57 5.54 5.05 5.01 5.00 4.98 4.60 4.59 4.58
3.82 3.80 3.79 3.79 3.78 3.77 3.76 3.75 3.75 3.74 3.73 3.72 3.71 3.70 3.05 3.03 3.01 2.99 2.66 2.62 2.61 2.58
O
SO2
N
CN
CN
3aa
2.12 2.30 3.96 1.29 1.00 1.97 1.97 1.00 0.99 1.00
8.5 8.0 7.5 7.0 6.5 6.0 5.5 5.0 4.5 4.0 3.5 3.0 2.5 2.0 1.5 1.0 0.5
f1 (ppm)

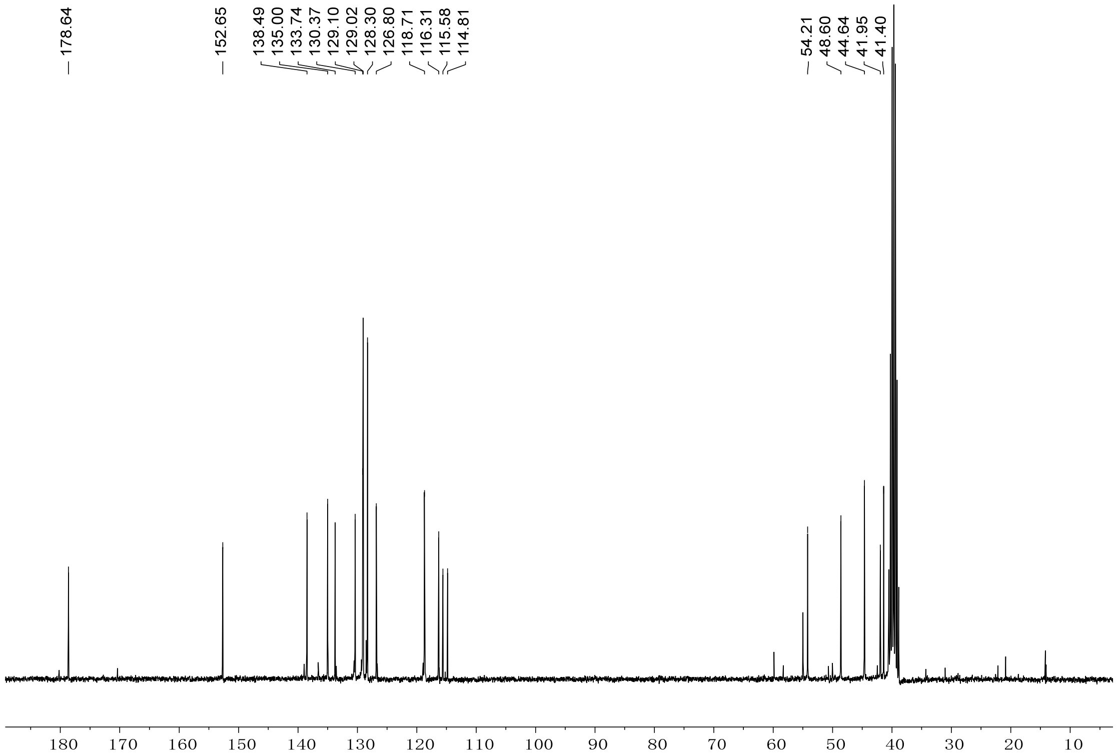
178.64
152.65
138.49 135.00 133.74 130.37 129.10 129.02 128.30 126.80 118.71 116.31 115.58 114.81
54.21 48.60 44.64 41.95 41.40
180 170 160 150 140 130 120 110 100 90 80 70 60 50 40 30 20 10
f1 (ppm)

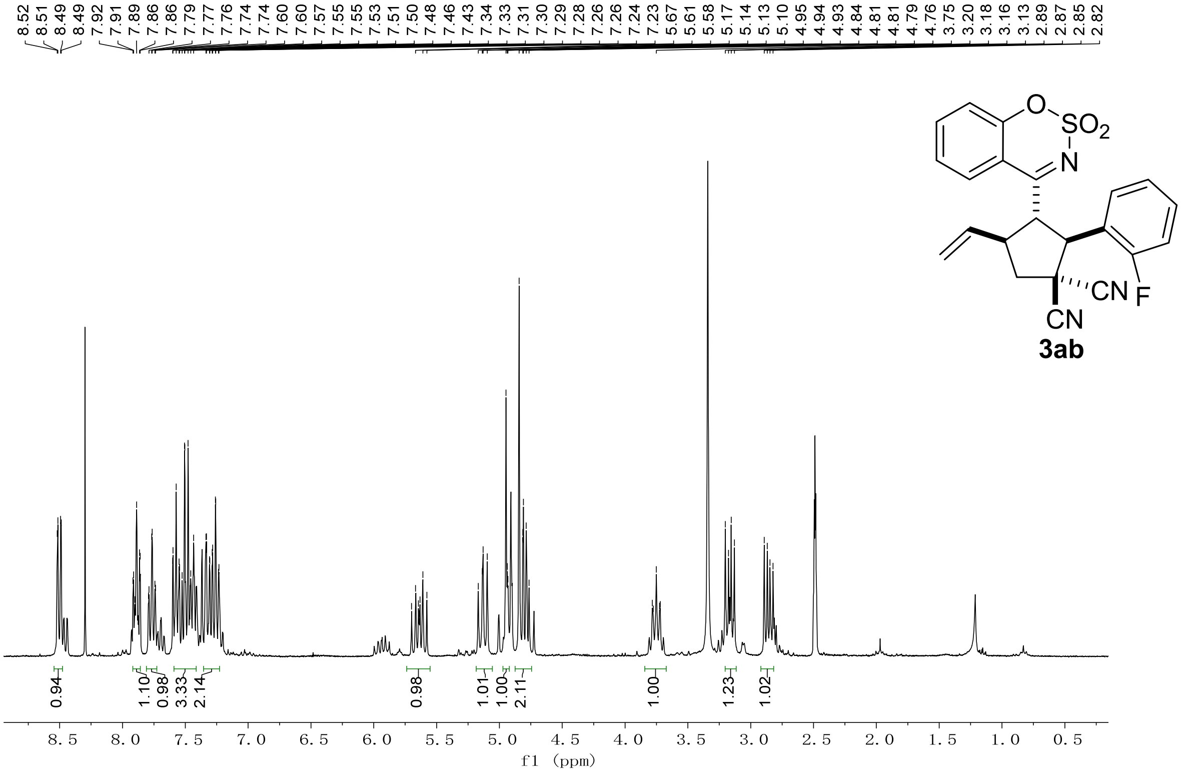
8.52 8.51 8.49 8.49 7.92 7.91 7.89 7.86 7.86 7.79 7.77 7.76 7.74 7.74 7.60 7.60 7.57 7.55 7.55 7.53 7.51 7.50 7.48 7.46 7.43 7.34 7.33 7.31 7.30 7.29 7.28 7.26 7.26 7.24 7.23 5.67 5.61 5.58 5.17 5.14 5.13 5.10 4.95 4.94 4.93 4.84 4.81 4.81 4.79 4.76 3.75 3.20 3.18 3.16 3.13 2.89 2.87 2.85 2.82
O
SO2
N
CN
F
CN
3ab
0.94 1.10 0.98 3.33 2.14 0.98 1.01 1.00 2.11 1.00 1.23 1.02
8.5 8.0 7.5 7.0 6.5 6.0 5.5 5.0 4.5 4.0 3.5 3.0 2.5 2.0 1.5 1.0 0.5
f1 (ppm)

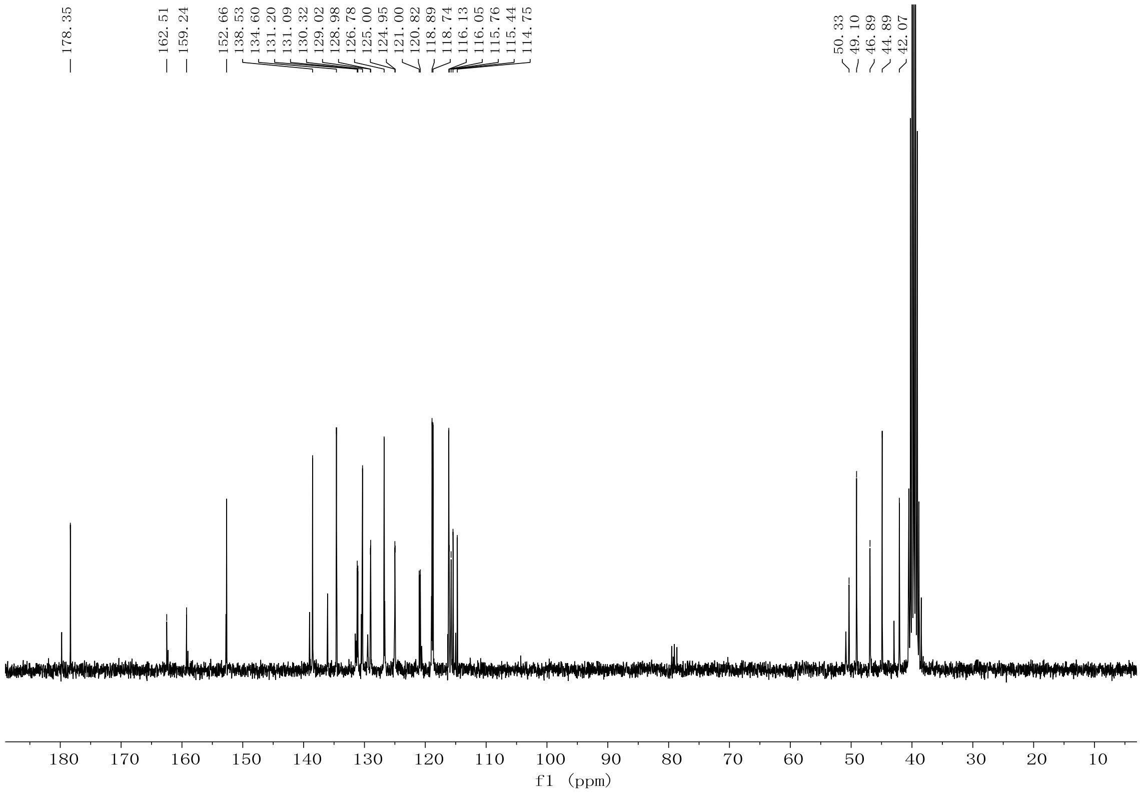
178.35 162.51 159.24 152.66 138.53 134.60 131.20 131.09 130.32 129.02 128.98 126.78 125.00 124.95 121.00 120.82 118.89 118.74 116.13 116.05 115.76 115.44 114.75
50.33 49.10 46.89 44.89 42.07
180 170 160 150 140 130 120 110 100 90 80 70 60 50 40 30 20 10
f1 (ppm)

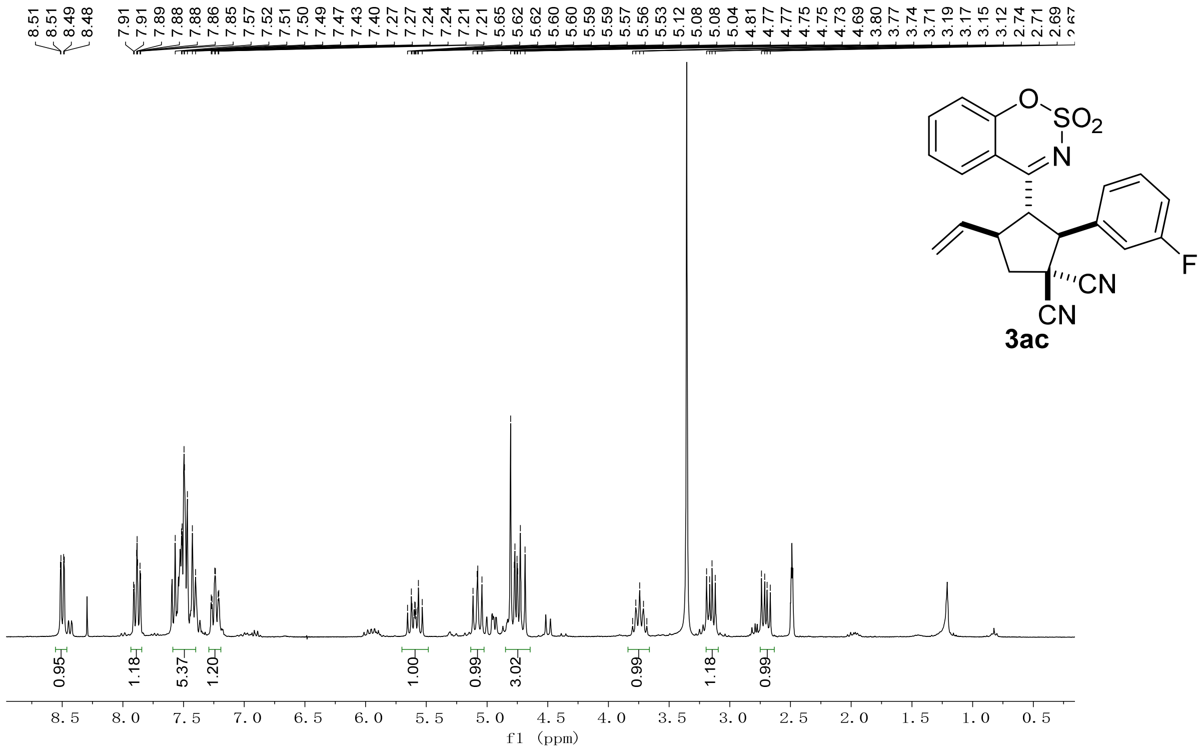
8.51
8.51
8.49
8.48
7.91
7.91
7.89
7.88
7.88
7.86
7.85
7.57
7.52
7.51
7.50
7.49
7.47
7.43
7.40
7.27
7.27
7.24
7.24
7.21
7.21
5.65
5.62
5.62
5.60
5.59
5.59
5.57
5.56
5.53
5.12
5.08
5.08
5.04
4.81
4.77
4.77
4.75
4.75
4.73
4.69
3.80
3.77
3.74
3.71
3.19
3.17
3.15
3.12
2.74
2.71
2.69
O
SO2
N
CN
CN
F
3ac
0.95
1.18
5.37
1.20
1.00
0.99
3.02
0.99
1.18
0.99
8.5 8.0 7.5 7.0 6.5 6.0 5.5 5.0 4.5 4.0 3.5 3.0 2.5 2.0 1.5 1.0 0.5
f1 (ppm)

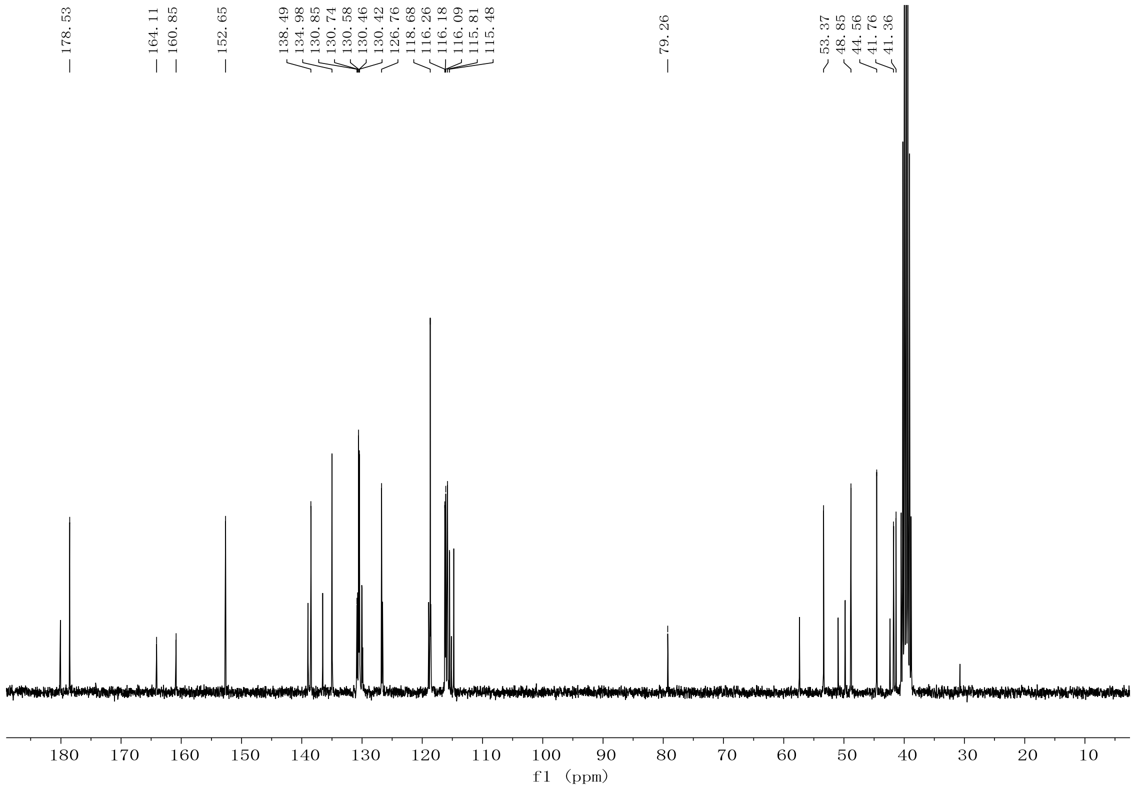
178.53
164.11
160.85
152.65
138.49
134.98
130.85
130.74
130.58
130.46
130.42
126.76
118.68
116.26
116.18
116.09
115.81
115.48
79.26
53.37
48.85
44.56
41.76
41.36
180 170 160 150 140 130 120 110 100 90 80 70 60 50 40 30 20 10
f1 (ppm)

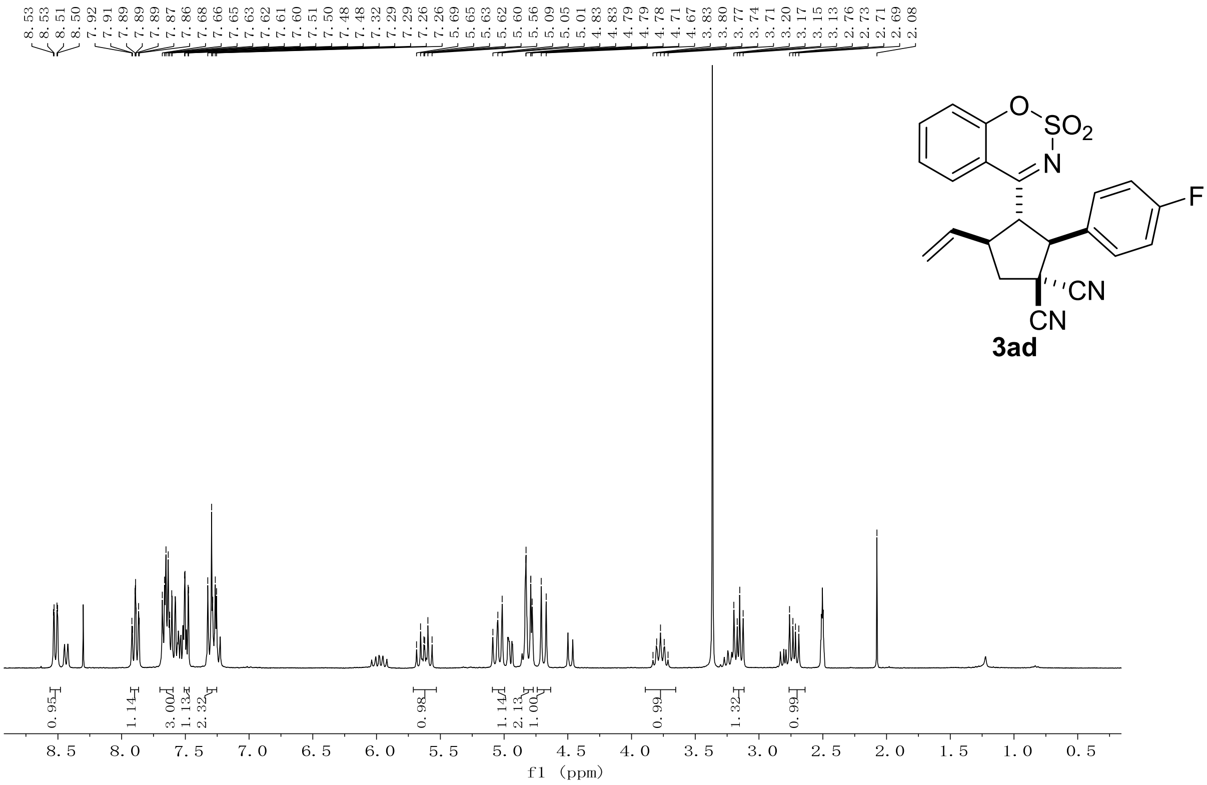
8.53 8.53 8.51 8.50 7.92 7.91 7.89 7.89 7.87 7.86 7.68 7.66 7.65 7.63 7.63 7.62 7.61 7.60 7.51 7.50 7.48 7.48 7.32 7.29 7.29 7.26 7.26 5.69 5.65 5.65 5.63 5.62 5.60 5.56 5.09 5.05 5.01 4.83 4.83 4.79 4.78 4.71 4.67 3.83 3.80 3.77 3.74 3.71 3.20 3.17 3.15 3.13 2.76 2.73 2.71 2.69 2.08
O
SO2
N
F
CN
CN
3ad
0.95 1.14 3.00 1.13 2.32 0.98 1.14 2.13 1.00 0.99 1.32 0.99
8.5 8.0 7.5 7.0 6.5 6.0 5.5 5.0 4.5 4.0 3.5 3.0 2.5 2.0 1.5 1.0 0.5
f1 (ppm)

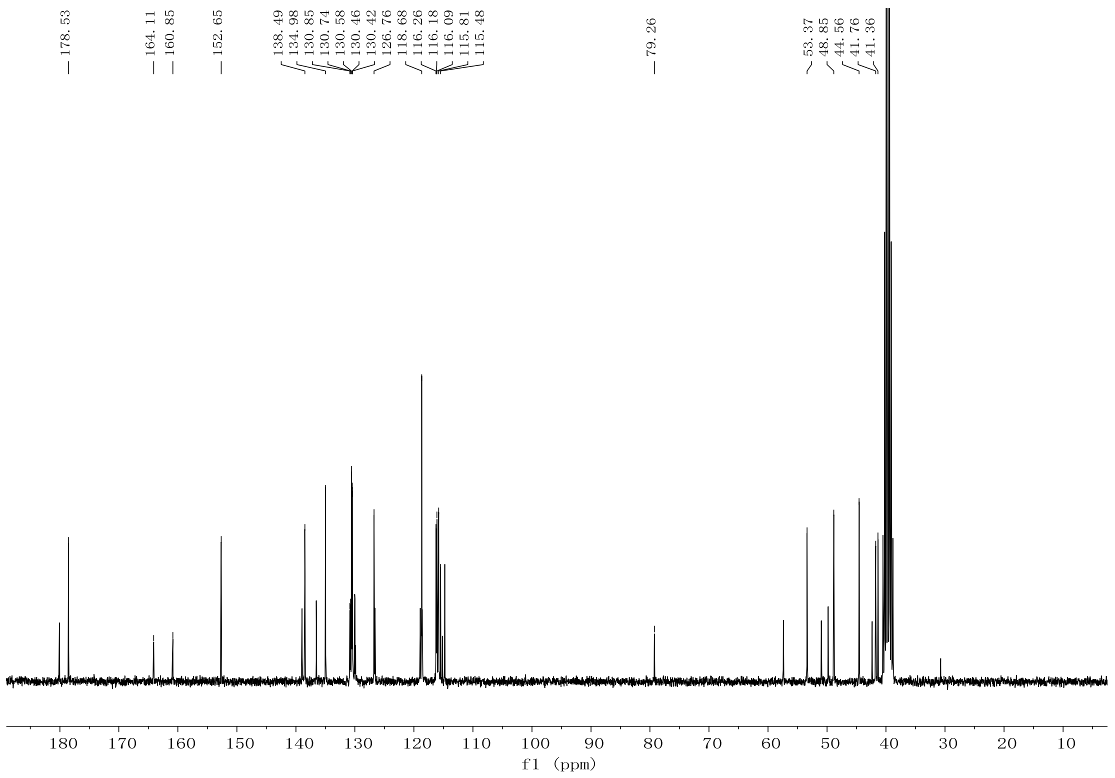
178.53 164.11 160.85 152.65 138.49 134.98 130.85 130.74 130.58 130.46 130.42 126.76 118.68 116.26 116.18 116.09 115.81 115.48 79.26 53.37 48.85 44.56 41.76 41.36
180 170 160 150 140 130 120 110 100 90 80 70 60 50 40 30 20 10
f1 (ppm)

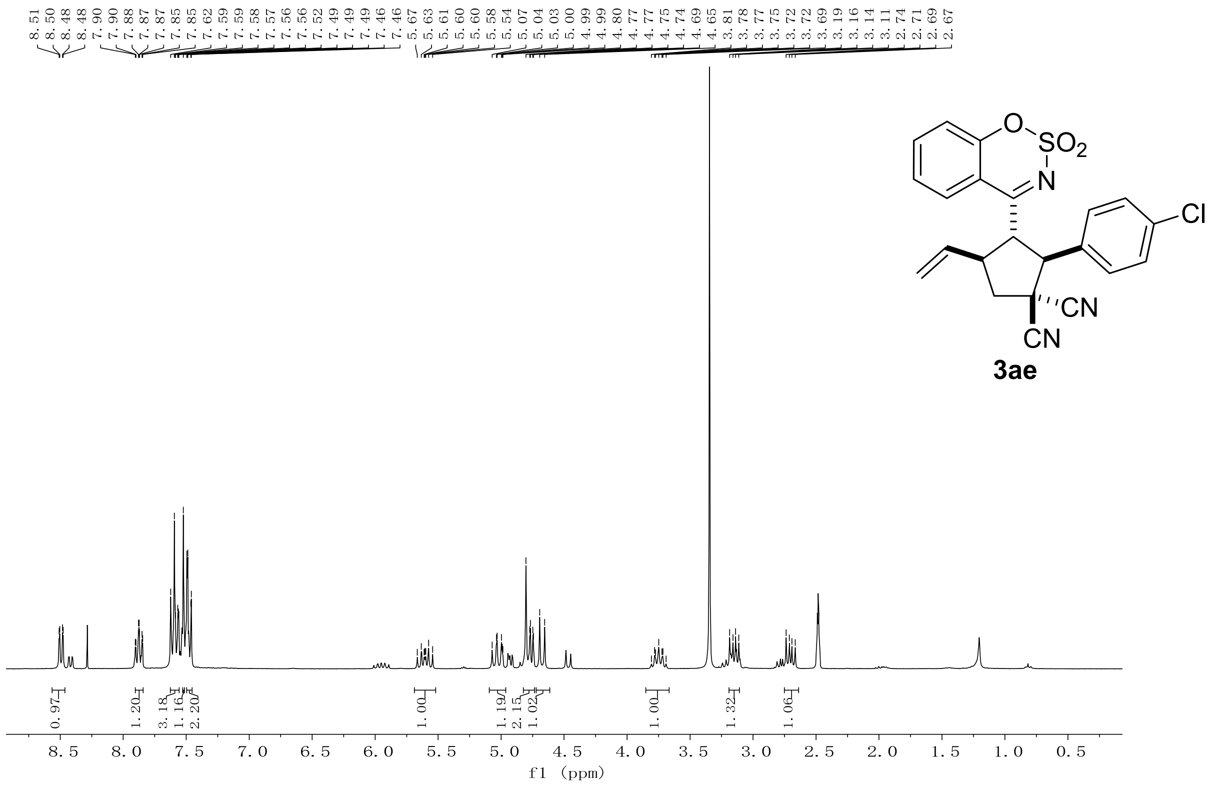
8.51 8.50 8.48 8.48 7.90 7.90 7.88 7.87 7.87 7.85 7.85 7.85 7.62 7.59 7.59 7.58 7.57 7.56 7.56 7.52 7.49 7.49 7.49 7.46 7.46 5.67 5.63 5.61 5.60 5.60 5.58 5.54 5.07 5.04 5.03 5.00 4.99 4.99 4.80 4.77 4.77 4.75 4.74 4.69 4.65 3.81 3.78 3.77 3.75 3.72 3.69 3.19 3.16 3.14 3.11 2.74 2.71 2.69 2.67
O
SO2
N
Cl
CN
CN
3ae
0.97 1.20 3.18 1.16 2.20 1.00 1.19 2.15 1.02 1.00 1.32 1.06
8.5 8.0 7.5 7.0 6.5 6.0 5.5 5.0 4.5 4.0 3.5 3.0 2.5 2.0 1.5 1.0 0.5
f1 (ppm)

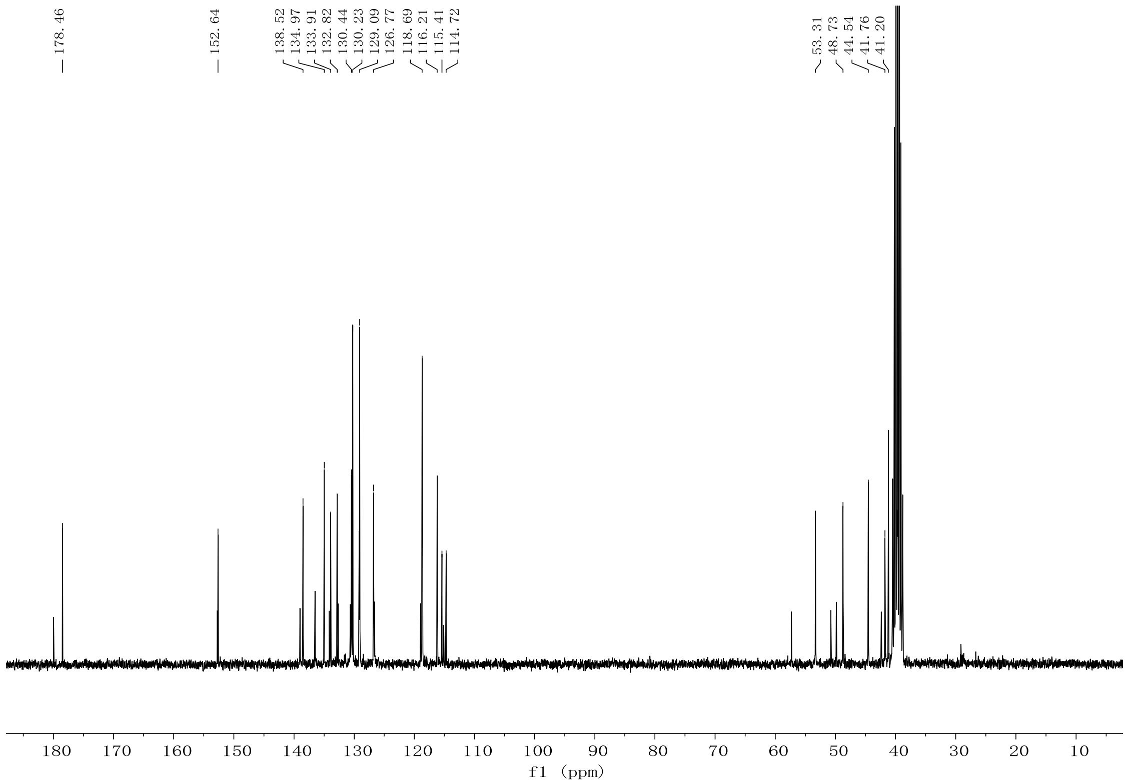
178.46
152.64
138.52 134.97 133.91 132.82 130.44 130.23 129.09 126.77 118.69 116.21 115.41 114.72
53.31 48.73 44.54 41.76 41.20
180 170 160 150 140 130 120 110 100 90 80 70 60 50 40 30 20 10
f1 (ppm)

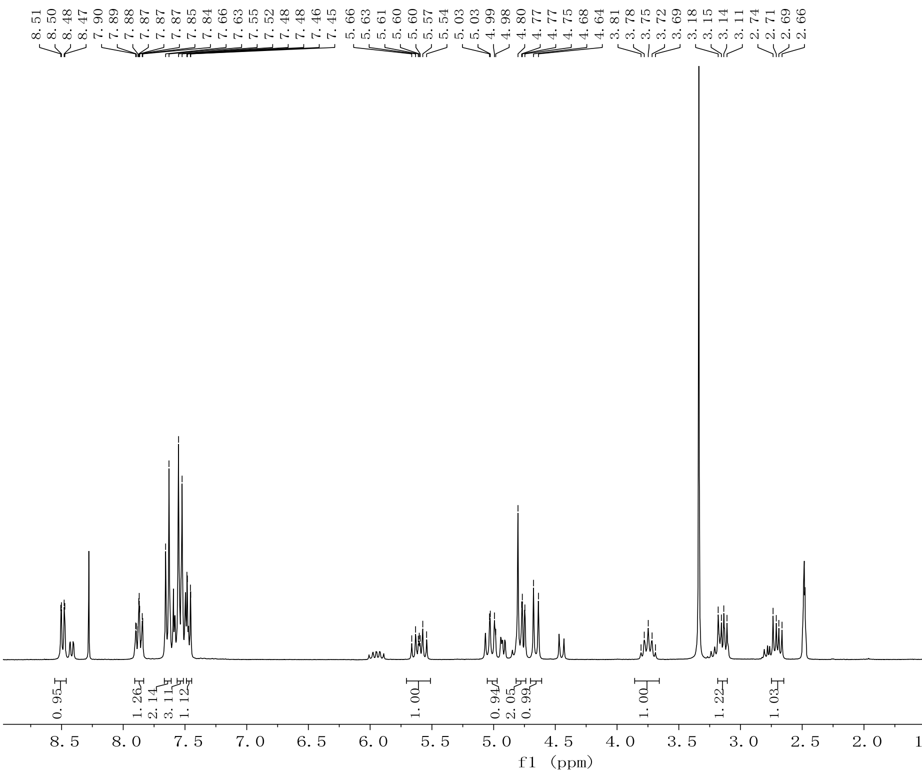
8.51
8.50
8.48
8.47
7.90
7.89
7.88
7.87
7.87
7.85
7.84
7.66
7.63
7.55
7.52
7.48
7.48
7.46
7.45
5.66
5.63
5.61
5.60
5.60
5.57
5.54
5.03
4.99
4.98
4.80
4.77
4.75
4.68
4.64
3.81
3.78
3.75
3.72
3.69
3.18
3.15
3.14
3.11
2.74
2.71
2.69
2.66
0.95
1.26
2.14
3.11
1.12
1.00
0.94
2.05
0.99
1.00
1.22
1.03
f1 (ppm)

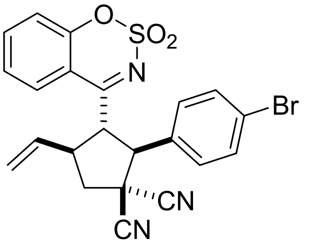
O
SO2
N
Br
CN
CN

3af

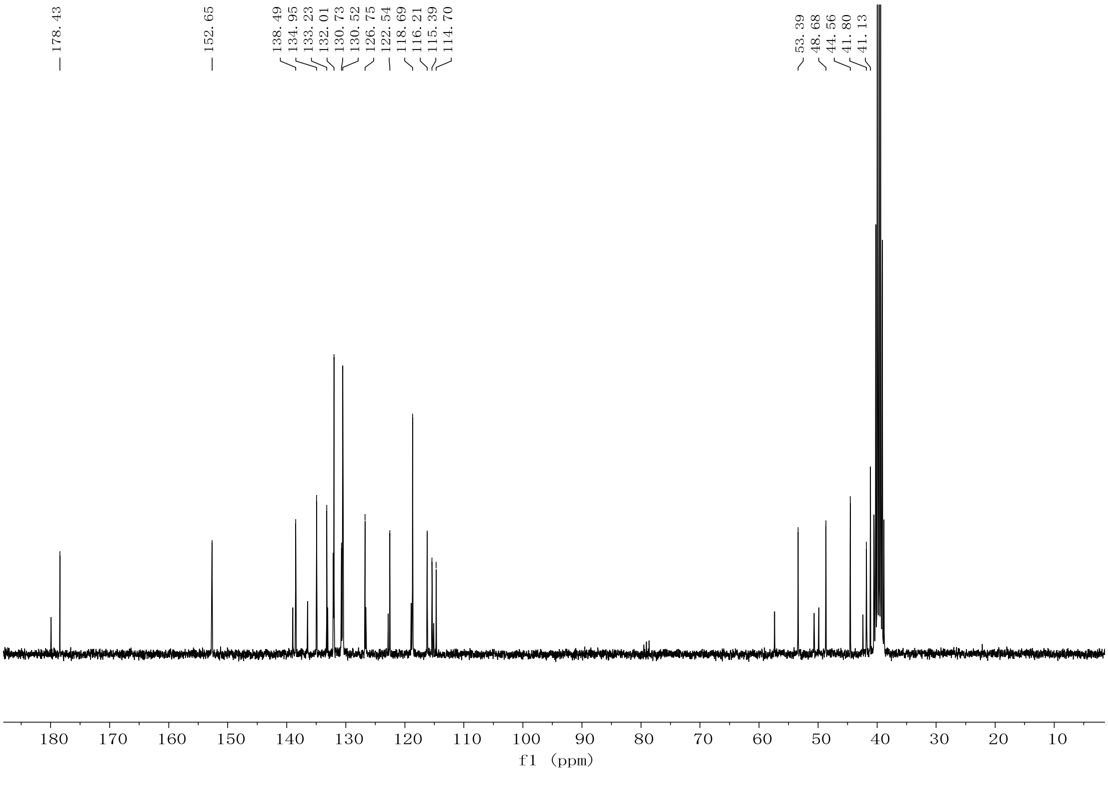
178.43
152.65
138.49
134.95
133.23
132.01
130.73
130.52
126.75
122.54
118.69
116.21
115.39
114.70
53.39
48.68
44.56
41.80
41.13
f1 (ppm)

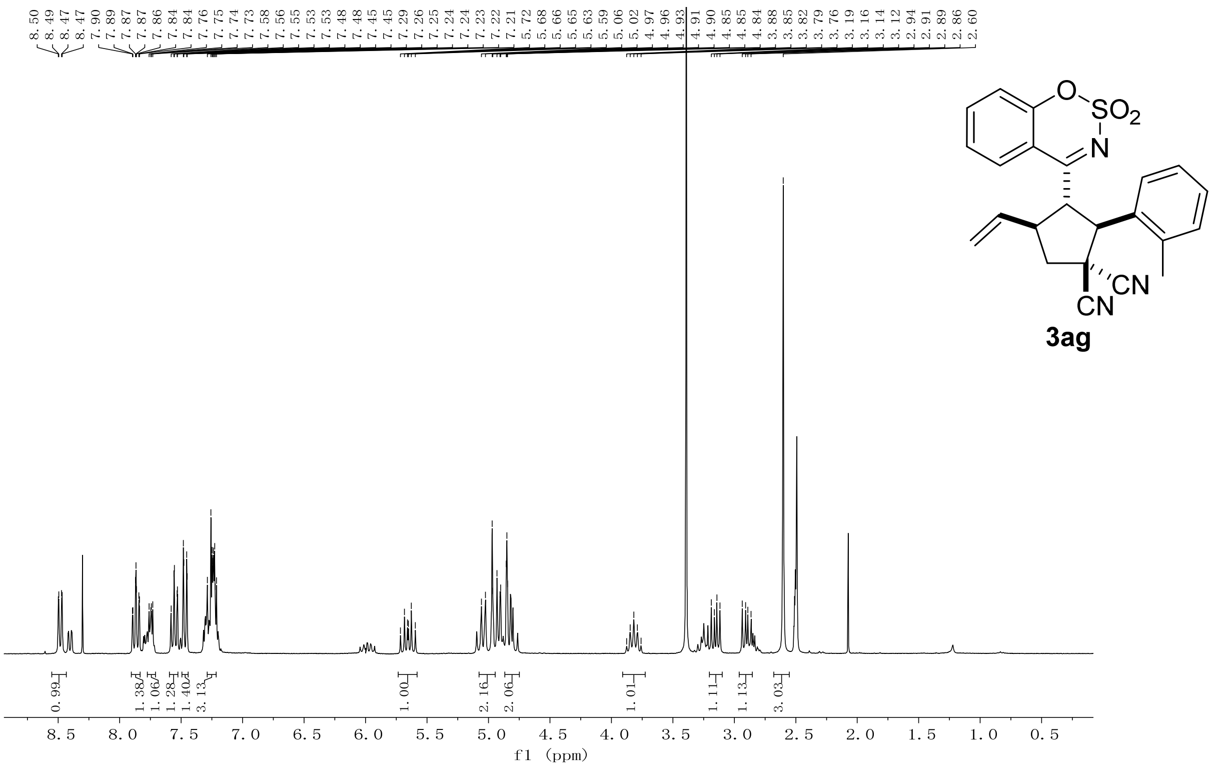
8.50 8.49 8.47 8.47 7.90 7.89 7.87 7.87 7.86 7.84 7.84 7.84 7.76 7.75 7.74 7.73 7.58 7.56 7.55 7.53 7.53 7.48 7.48 7.45 7.45 7.29 7.26 7.25 7.24 7.24 7.23 7.22 7.21 5.72 5.68 5.66 5.65 5.63 5.59 5.06 5.02 4.97 4.96 4.93 4.91 4.90 4.85 4.85 4.84 3.88 3.85 3.82 3.79 3.76 3.19 3.16 3.14 3.12 2.94 2.91 2.89 2.86 2.60
0.99 1.38 1.06 1.28 1.40 3.13 1.00 2.16 2.06 1.01 1.11 1.13 3.03
8.5 8.0 7.5 7.0 6.5 6.0 5.5 5.0 4.5 4.0 3.5 3.0 2.5 2.0 1.5 1.0 0.5
f1 (ppm)
O
SO2
N
CN
CN
3ag

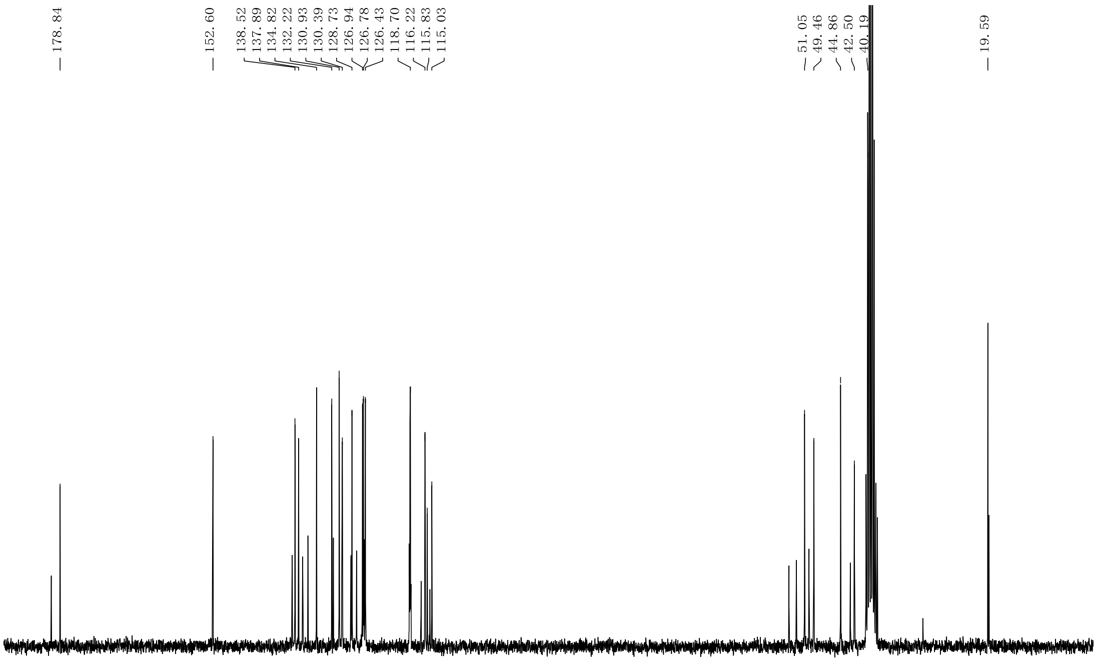
178.84
152.60
138.52 137.89 134.82 132.22 130.93 130.39 128.73 126.94 126.78 126.43 118.70 116.22 115.83 115.03
51.05 49.46 44.86 42.50 40.19
19.59
180 170 160 150 140 130 120 110 100 90 80 70 60 50 40 30 20 10
f1 (ppm)

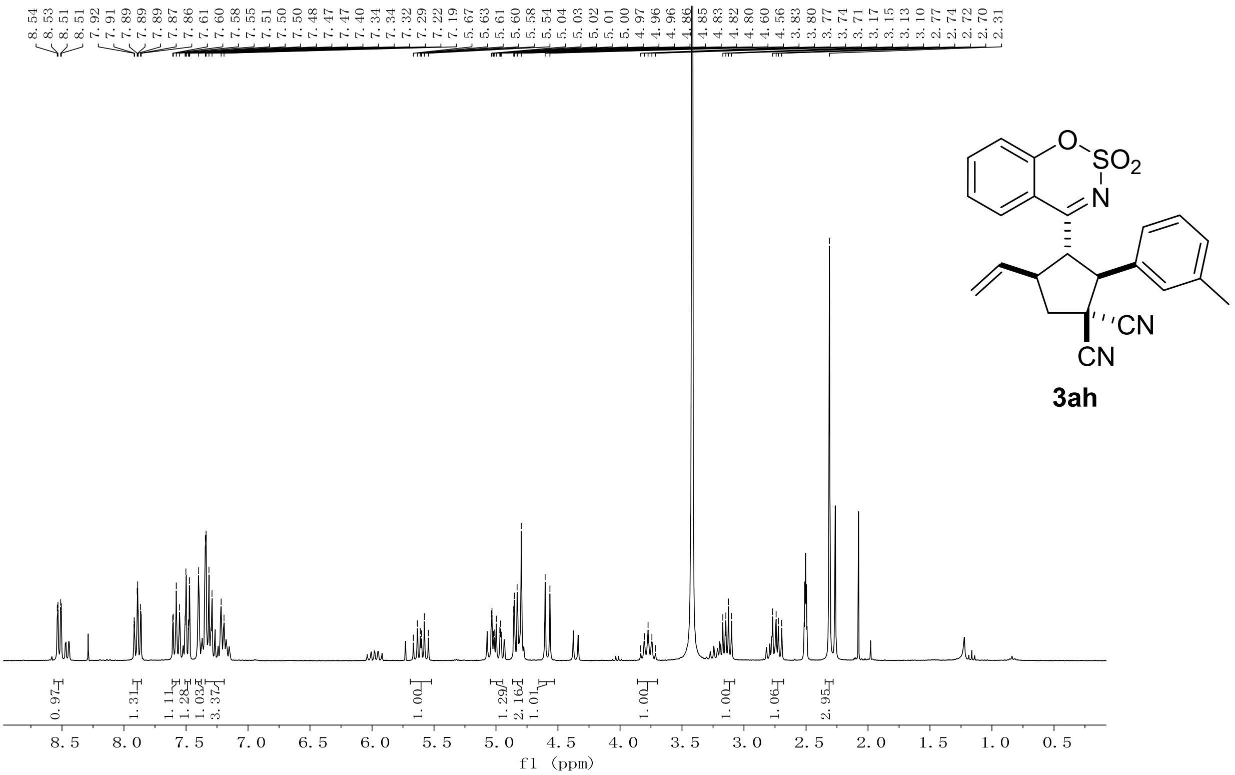
8.54
8.53
8.51
8.51
7.92
7.91
7.89
7.89
7.89
7.87
7.86
7.61
7.60
7.58
7.55
7.51
7.50
7.48
7.47
7.40
7.34
7.34
7.32
7.29
7.22
7.19
5.67
5.63
5.61
5.60
5.58
5.54
5.04
5.03
5.02
5.01
5.00
4.97
4.96
4.96
4.86
4.85
4.83
4.82
4.80
4.60
4.56
3.83
3.80
3.77
3.74
3.71
3.17
3.15
3.13
3.10
2.77
2.74
2.72
2.70
2.31
0.97
1.31
1.11
1.28
1.03
3.37
1.00
1.29
2.16
1.01
1.00
1.00
1.06
2.95
8.5 8.0 7.5 7.0 6.5 6.0 5.5 5.0 4.5 4.0 3.5 3.0 2.5 2.0 1.5 1.0 0.5
f1 (ppm)
O
SO2
N
CN
CN

3ah

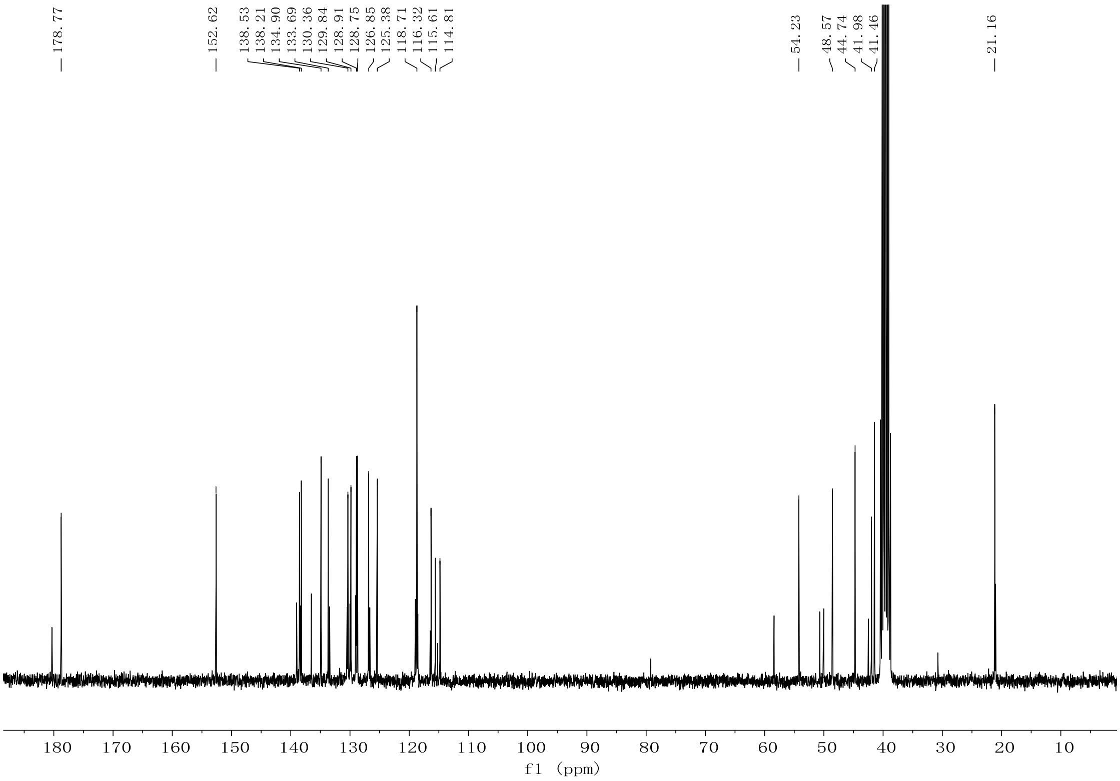
178.77
152.62
138.53
138.21
134.90
133.69
130.36
129.84
128.91
128.75
126.85
125.38
118.71
116.32
115.61
114.81
54.23
48.57
44.74
41.98
41.46
21.16
180 170 160 150 140 130 120 110 100 90 80 70 60 50 40 30 20 10
f1 (ppm)

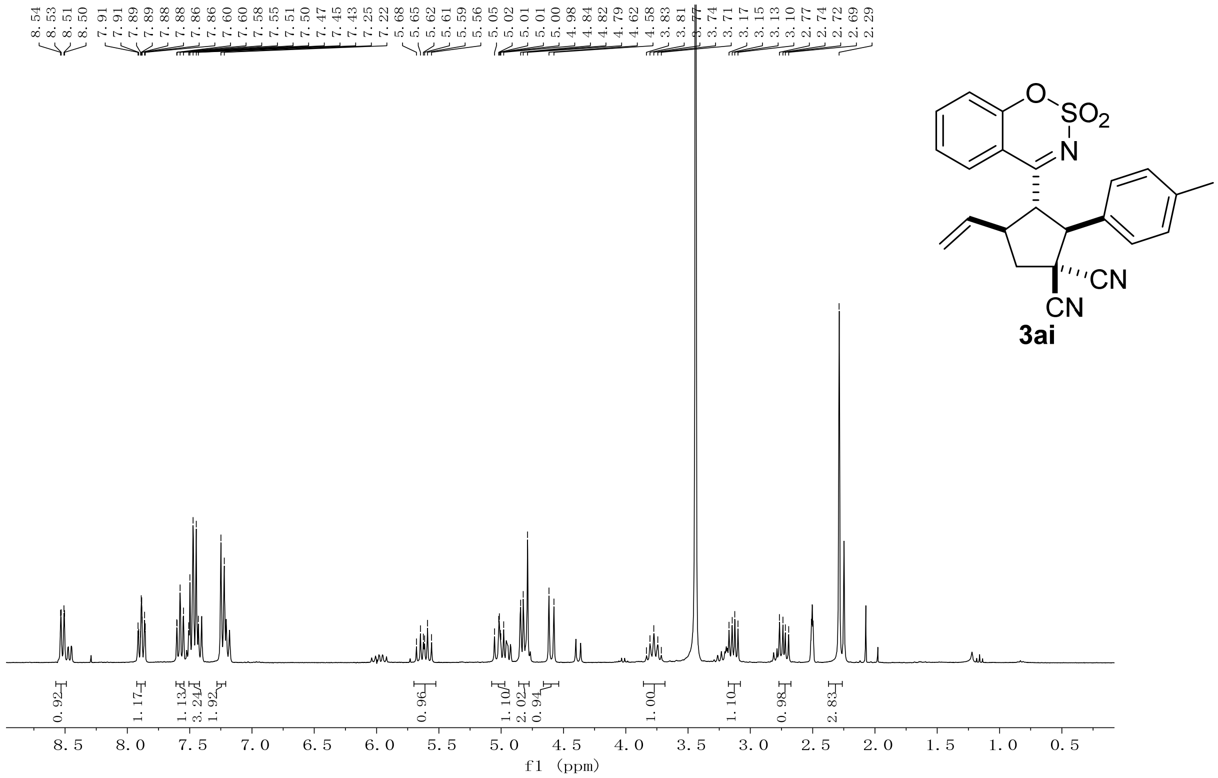
8.54 8.53 8.51 8.50 7.91 7.91 7.89 7.89 7.88 7.88 7.86 7.86 7.60 7.58 7.55 7.51 7.50 7.47 7.45 7.43 7.25 7.22 5.68 5.65 5.62 5.61 5.59 5.56 5.05 5.02 5.01 5.01 5.00 4.98 4.84 4.82 4.79 4.62 4.58 3.83 3.81 3.77 3.74 3.71 3.17 3.15 3.13 3.10 2.77 2.74 2.72 2.69 2.29
0.92 1.17 1.13 3.24 1.92 0.96 1.10 2.02 0.94 1.00 1.10 0.98 2.83
8.5 8.0 7.5 7.0 6.5 6.0 5.5 5.0 4.5 4.0 3.5 3.0 2.5 2.0 1.5 1.0 0.5
f1 (ppm)
O
SO2
N
CN
CN

3ai

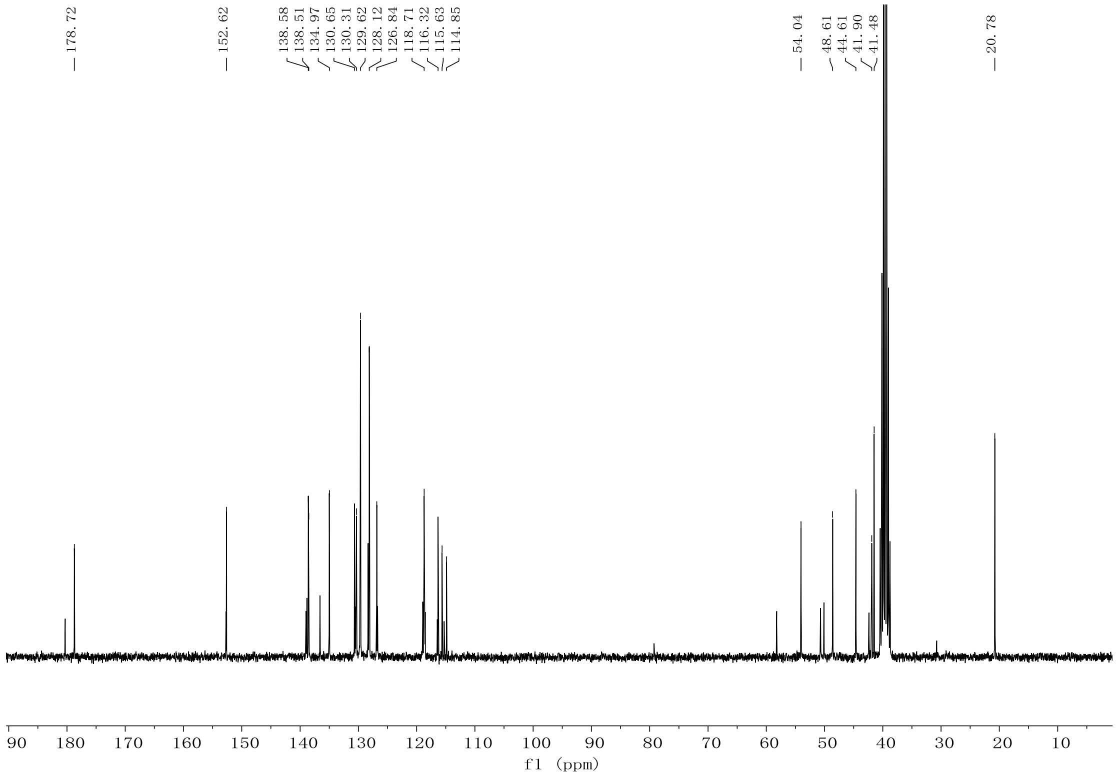
178.72 152.62 138.58 138.51 134.97 130.65 130.31 129.62 128.12 126.84 118.71 116.32 115.63 114.85 54.04 48.61 44.61 41.90 41.48 20.78
190 180 170 160 150 140 130 120 110 100 90 80 70 60 50 40 30 20 10
f1 (ppm)

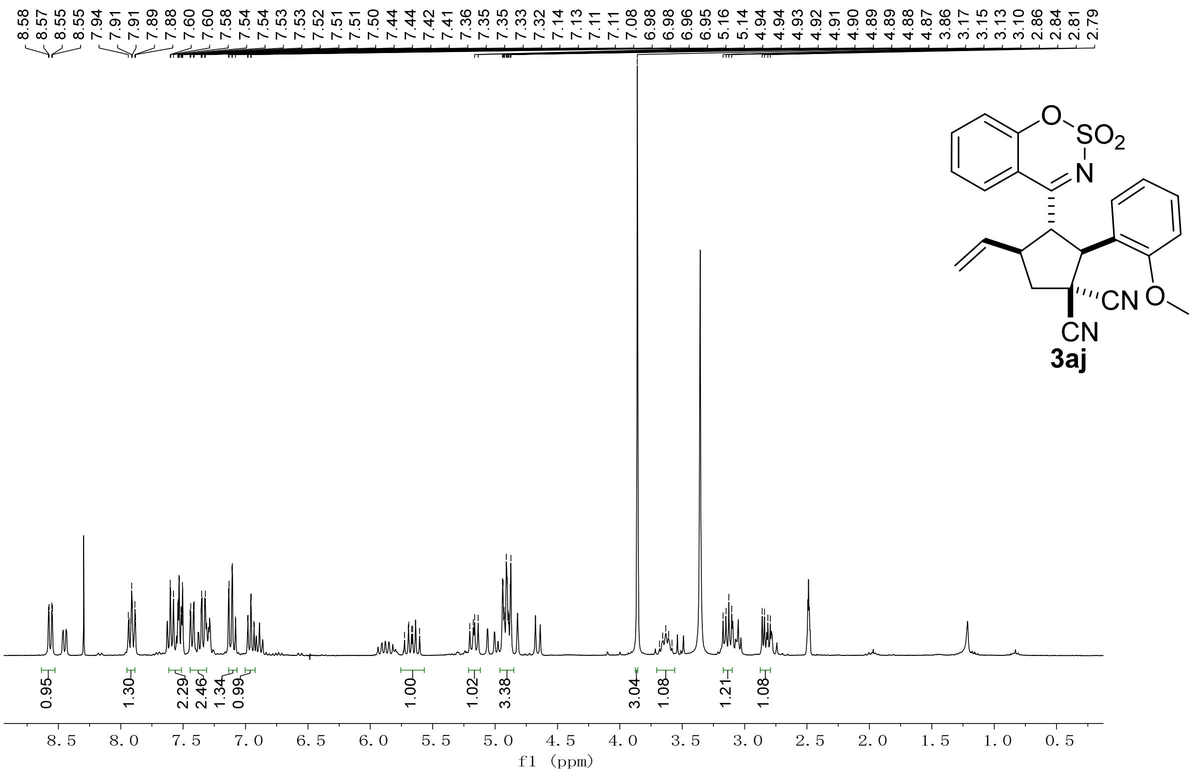
8.58
8.57
8.55
8.55
7.94
7.91
7.91
7.89
7.88
7.60
7.60
7.58
7.54
7.54
7.53
7.53
7.52
7.51
7.51
7.50
7.44
7.44
7.42
7.41
7.36
7.35
7.35
7.33
7.32
7.14
7.13
7.11
7.11
7.08
6.98
6.98
6.96
6.95
5.16
5.14
4.94
4.94
4.93
4.92
4.91
4.90
4.89
4.89
4.88
4.87
3.86
3.17
3.15
3.13
3.10
2.86
2.84
2.81
2.79
O
SO2
N
CN
O
CN
3aj
0.95
1.30
2.29
2.46
1.34
0.99
1.00
1.02
3.38
3.04
1.08
1.21
1.08
8.5 8.0 7.5 7.0 6.5 6.0 5.5 5.0 4.5 4.0 3.5 3.0 2.5 2.0 1.5 1.0 0.5
f1 (ppm)

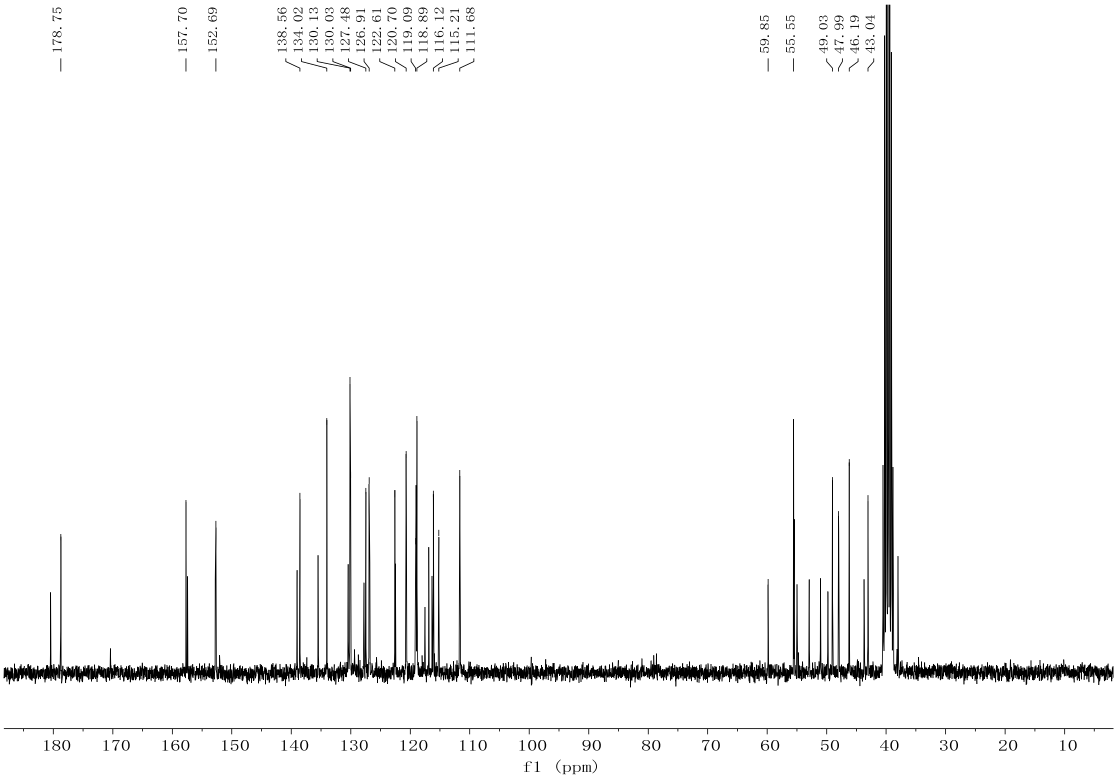
178.75
157.70
152.69
138.56
134.02
130.13
130.03
127.48
126.91
122.61
120.70
119.09
118.89
116.12
115.21
111.68
59.85
55.55
49.03
47.99
46.19
43.04
180 170 160 150 140 130 120 110 100 90 80 70 60 50 40 30 20 10
f1 (ppm)

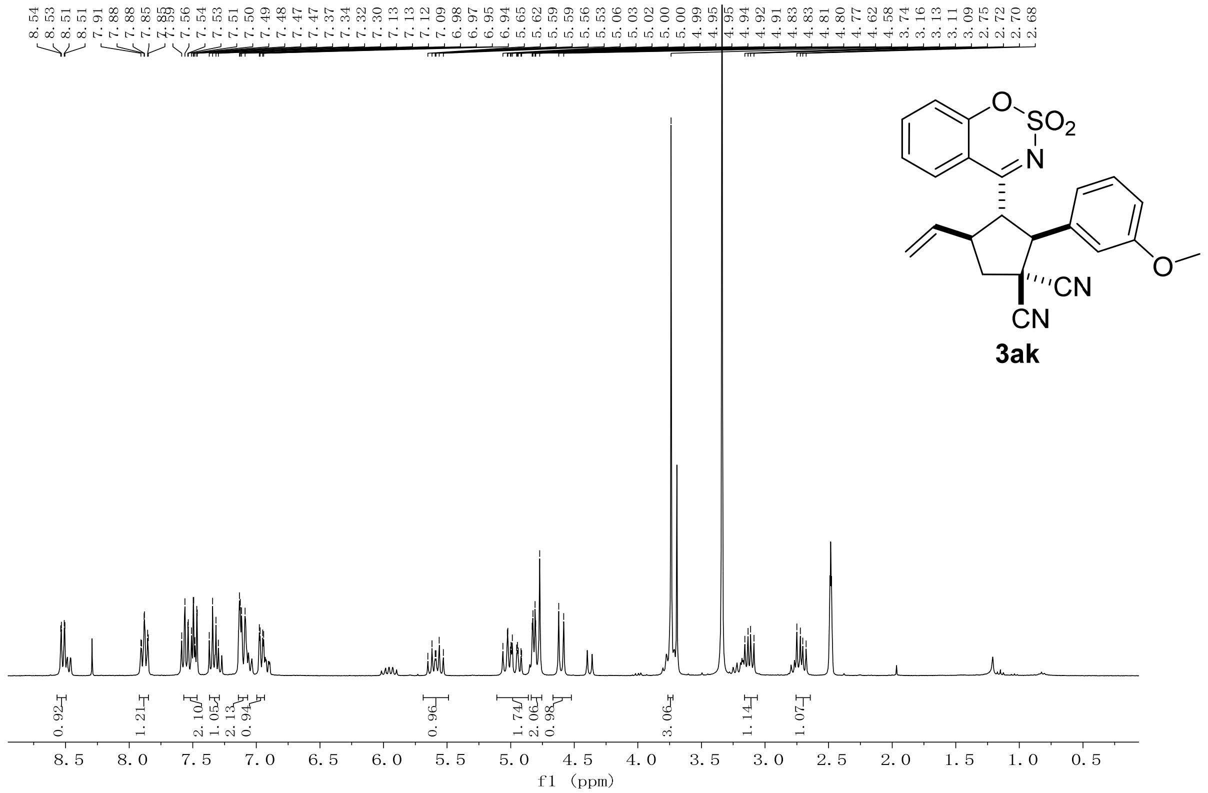
8.54 8.53 8.51 8.51 7.91 7.88 7.85 7.85 7.59 7.56 7.54 7.53 7.51 7.50 7.49 7.48 7.47 7.47 7.37 7.34 7.32 7.30 7.13 7.13 7.12 7.09 6.98 6.97 6.95 6.94 5.65 5.62 5.59 5.59 5.56 5.53 5.06 5.03 5.02 5.00 5.00 4.99 4.95 4.95 4.94 4.92 4.91 4.83 4.83 4.81 4.80 4.77 4.62 4.58 3.74 3.16 3.13 3.11 3.09 2.75 2.72 2.70 2.68
O
SO2
N
O
CN
CN
3ak
0.92 1.21 2.10 1.05 2.13 0.94 0.96 1.74 2.06 0.98 3.06 1.14 1.07
8.5 8.0 7.5 7.0 6.5 6.0 5.5 5.0 4.5 4.0 3.5 3.0 2.5 2.0 1.5 1.0 0.5
f1 (ppm)

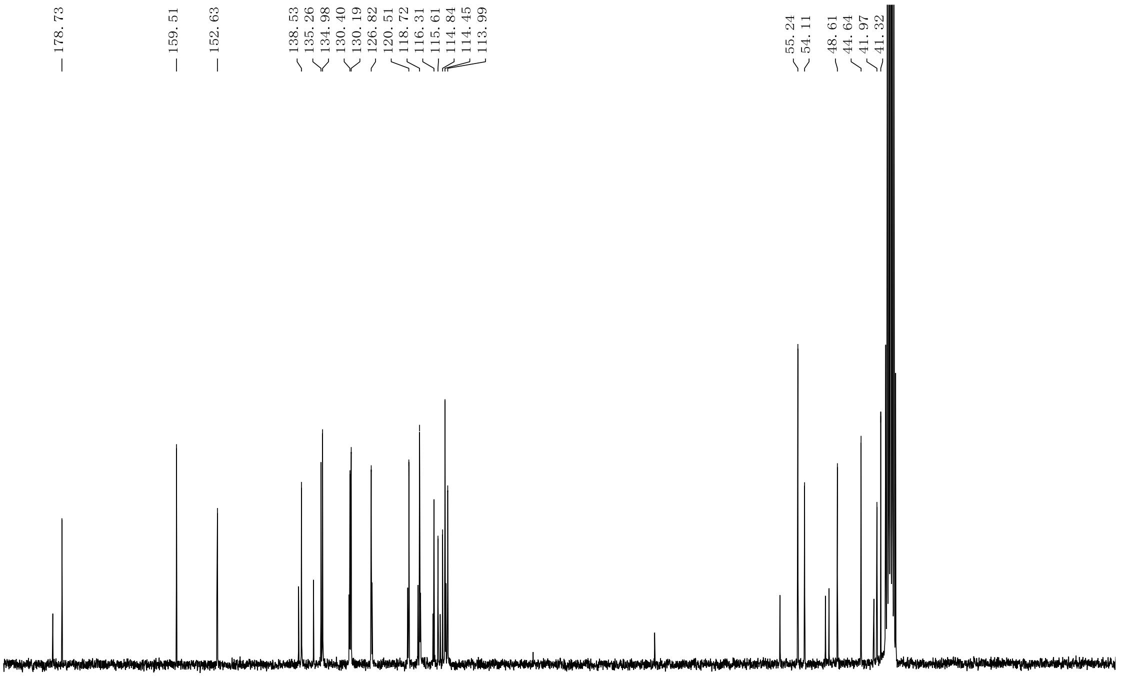
178.73 159.51 152.63 138.53 135.26 134.98 130.40 130.19 126.82 120.51 118.72 116.31 115.61 114.84 114.45 113.99 55.24 54.11 48.61 44.64 41.97 41.32
180 170 160 150 140 130 120 110 100 90 80 70 60 50 40 30 20 10
f1 (ppm)

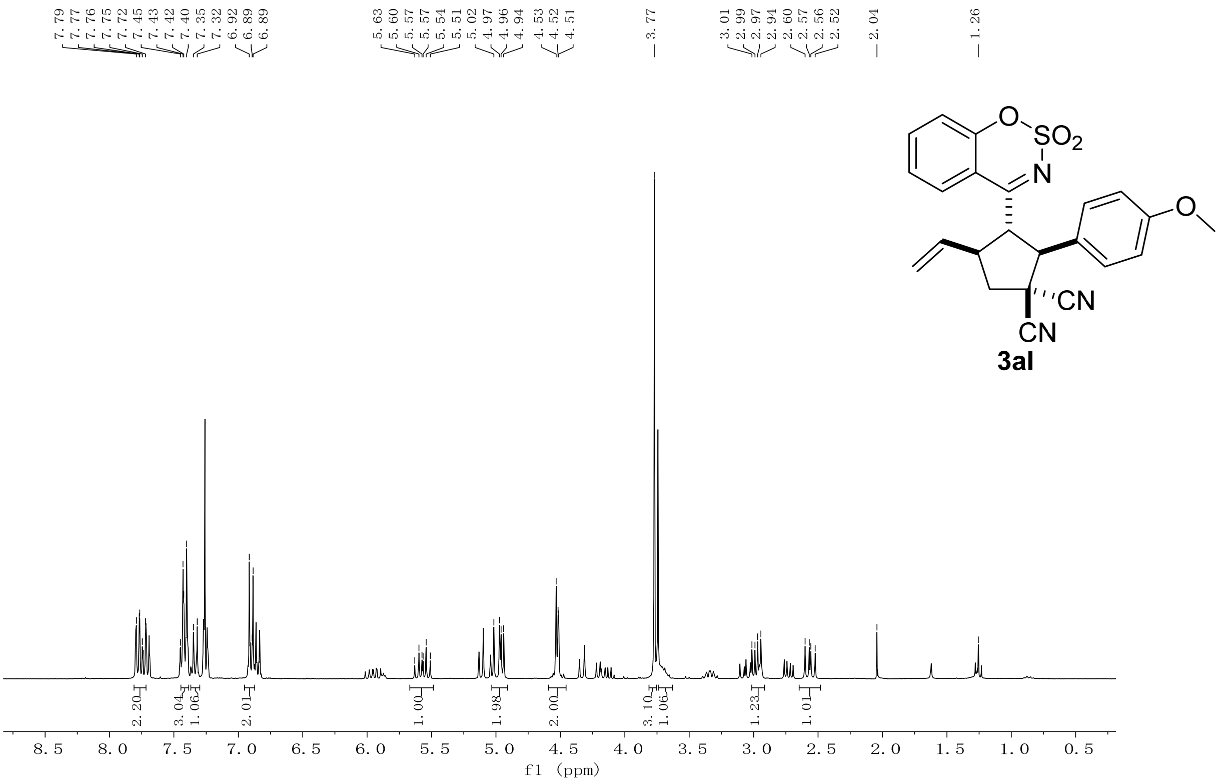
7.79 7.77 7.76 7.75 7.72 7.45 7.43 7.42 7.40 7.35 7.32 6.92 6.89 6.89
5.63 5.60 5.57 5.57 5.54 5.51 5.02 4.97 4.96 4.94 4.53 4.52 4.51
3.77
3.01 2.99 2.97 2.94 2.60 2.57 2.56 2.52
2.04
1.26
O
SO2
N
CN
CN
3al
2.20 3.04 1.06 2.01 1.00 1.98 2.00 3.10 1.06 1.23 1.01
8.5 8.0 7.5 7.0 6.5 6.0 5.5 5.0 4.5 4.0 3.5 3.0 2.5 2.0 1.5 1.0 0.5
f1 (ppm)

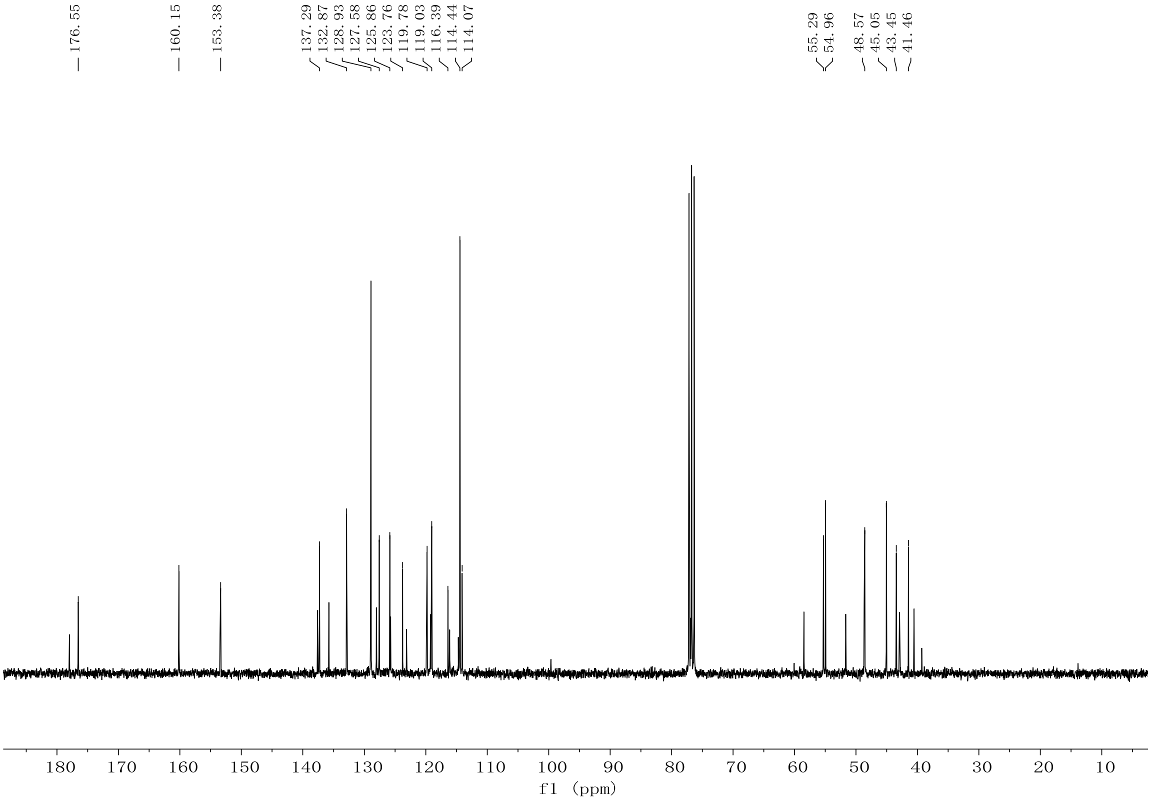
176.55
160.15
153.38
137.29 132.87 128.93 127.58 125.86 123.76 119.78 119.03 116.39 114.44 114.07
55.29 54.96
48.57 45.05 43.45 41.46
180 170 160 150 140 130 120 110 100 90 80 70 60 50 40 30 20 10
f1 (ppm)

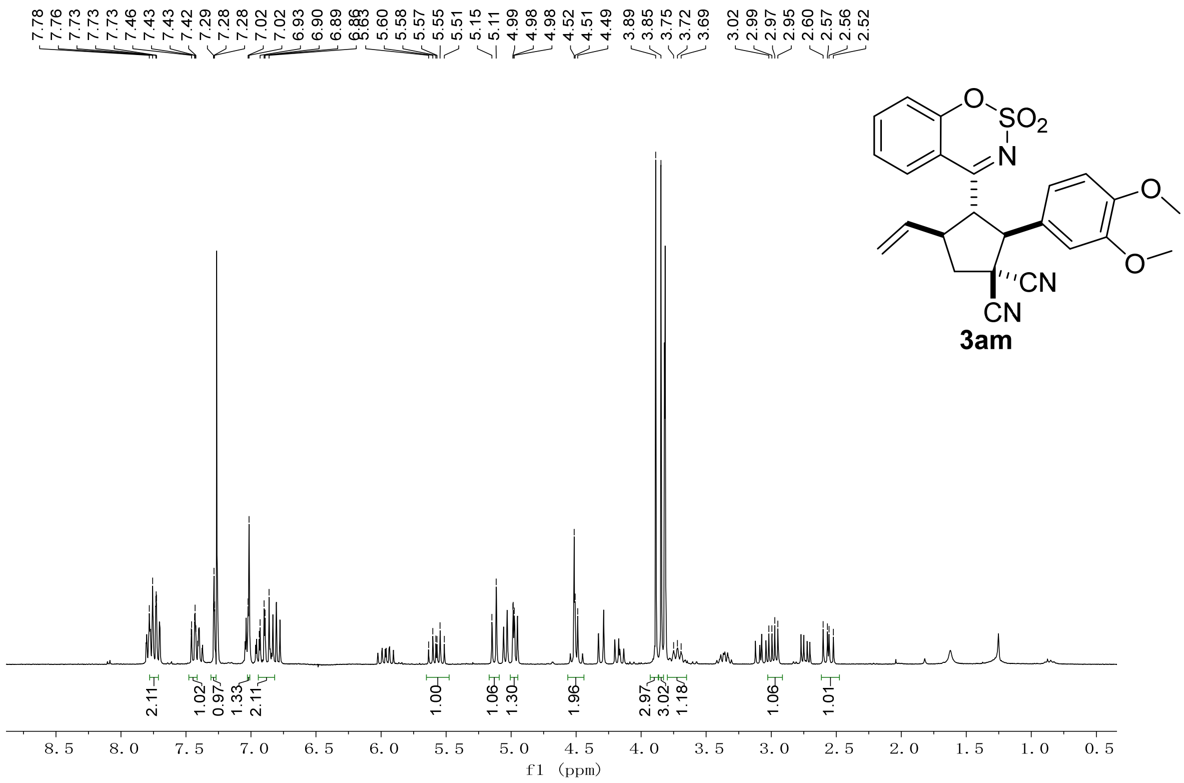
7.78 7.76 7.73 7.73 7.73 7.46 7.43 7.43 7.42 7.29 7.28 7.28 7.02 7.02 6.93 6.90 6.89 6.86 5.63 5.60 5.58 5.57 5.55 5.51 5.15 5.11 4.99 4.98 4.98 4.52 4.51 4.49 3.89 3.85 3.75 3.72 3.69 3.02 2.99 2.97 2.95 2.60 2.57 2.56 2.52
O
SO2
N
O
O
CN
CN
3am
2.11 1.02 0.97 1.33 2.11 1.00 1.06 1.30 1.96 2.97 3.02 1.18 1.06 1.01
8.5 8.0 7.5 7.0 6.5 6.0 5.5 5.0 4.5 4.0 3.5 3.0 2.5 2.0 1.5 1.0 0.5
f1 (ppm)

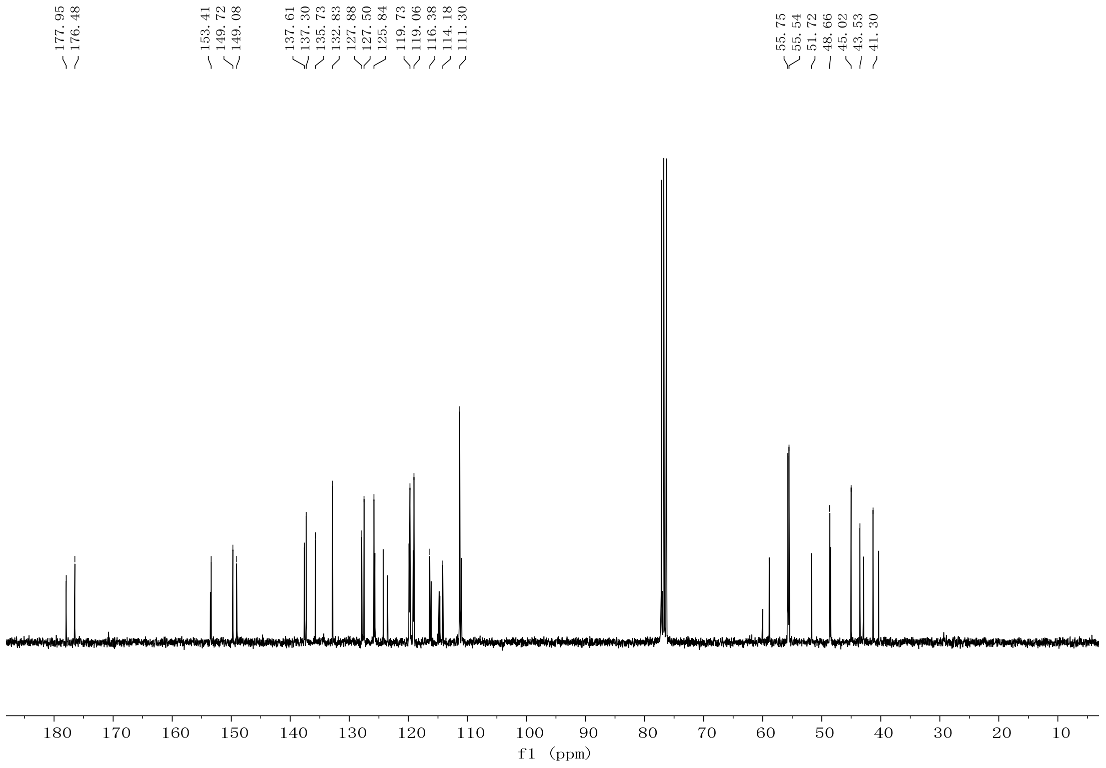
177.95 176.48 153.41 149.72 149.08 137.61 137.30 135.73 132.83 127.88 127.50 125.84 119.73 119.06 116.38 114.18 111.30 55.75 55.54 51.72 48.66 45.02 43.53 41.30
180 170 160 150 140 130 120 110 100 90 80 70 60 50 40 30 20 10
f1 (ppm)

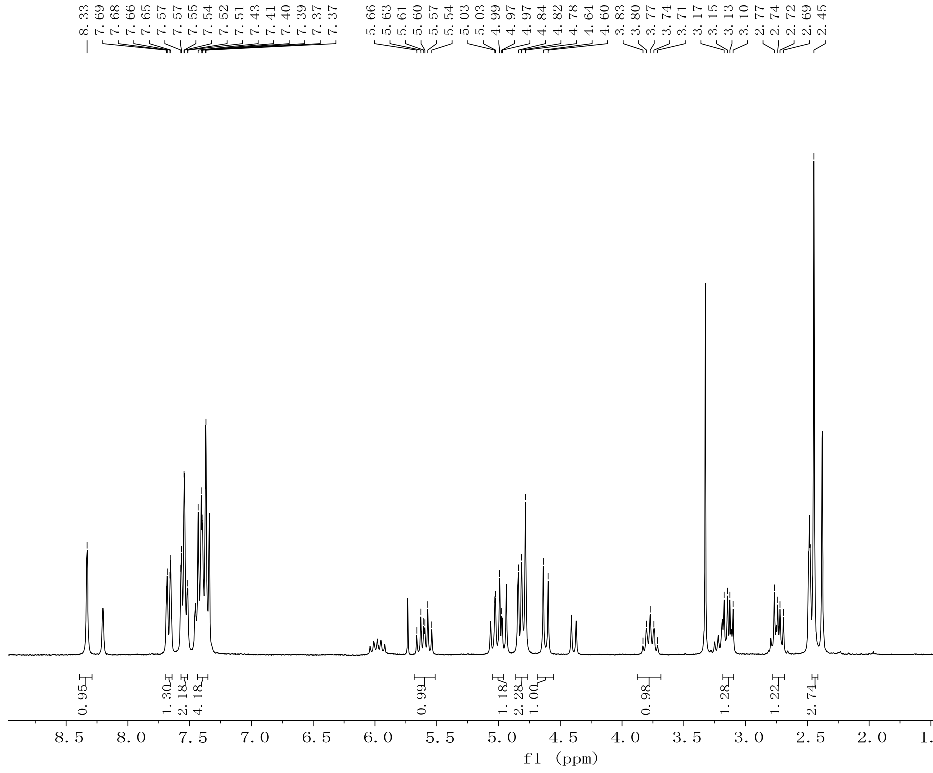

8.33
7.69
7.68
7.66
7.65
7.57
7.57
7.55
7.54
7.52
7.51
7.43
7.41
7.40
7.39
7.37
7.37
5.66
5.63
5.61
5.60
5.57
5.54
5.03
5.03
4.99
4.97
4.97
4.84
4.82
4.78
4.64
4.60
3.83
3.80
3.77
3.74
3.71
3.17
3.15
3.13
3.10
2.77
2.74
2.72
2.69
2.45
0.95
1.30
2.18
4.18
0.99
1.18
2.28
1.00
0.98
1.28
1.22
2.74
8.5 8.0 7.5 7.0 6.5 6.0 5.5 5.0 4.5 4.0 3.5 3.0 2.5 2.0 1.5 1.0 0.5
f1 (ppm)

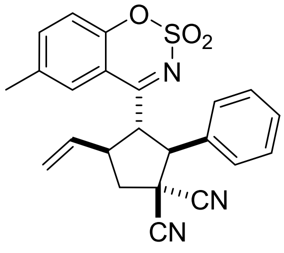

O
SO2
N
CN
CN

3an

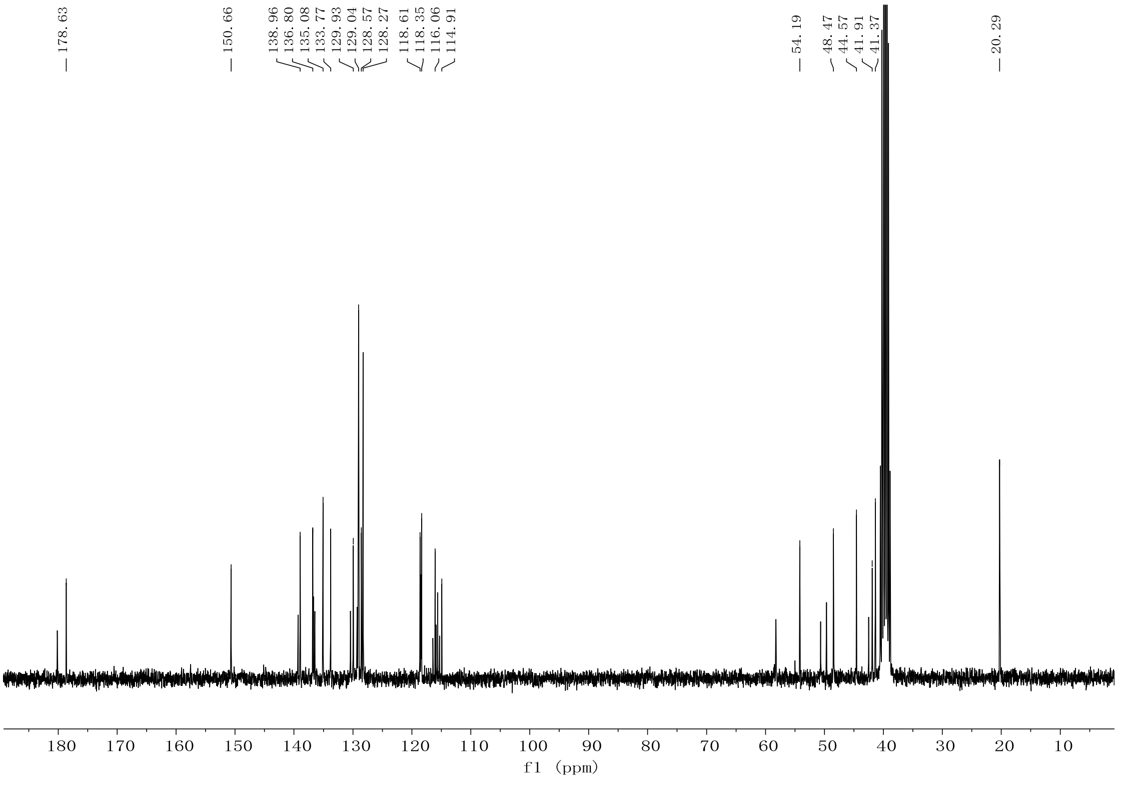

178.63
150.66
138.96
136.80
135.08
133.77
129.93
129.04
128.57
128.27
118.61
118.35
116.06
114.91
54.19
48.47
44.57
41.91
41.37
20.29
180 170 160 150 140 130 120 110 100 90 80 70 60 50 40 30 20 10
f1 (ppm)

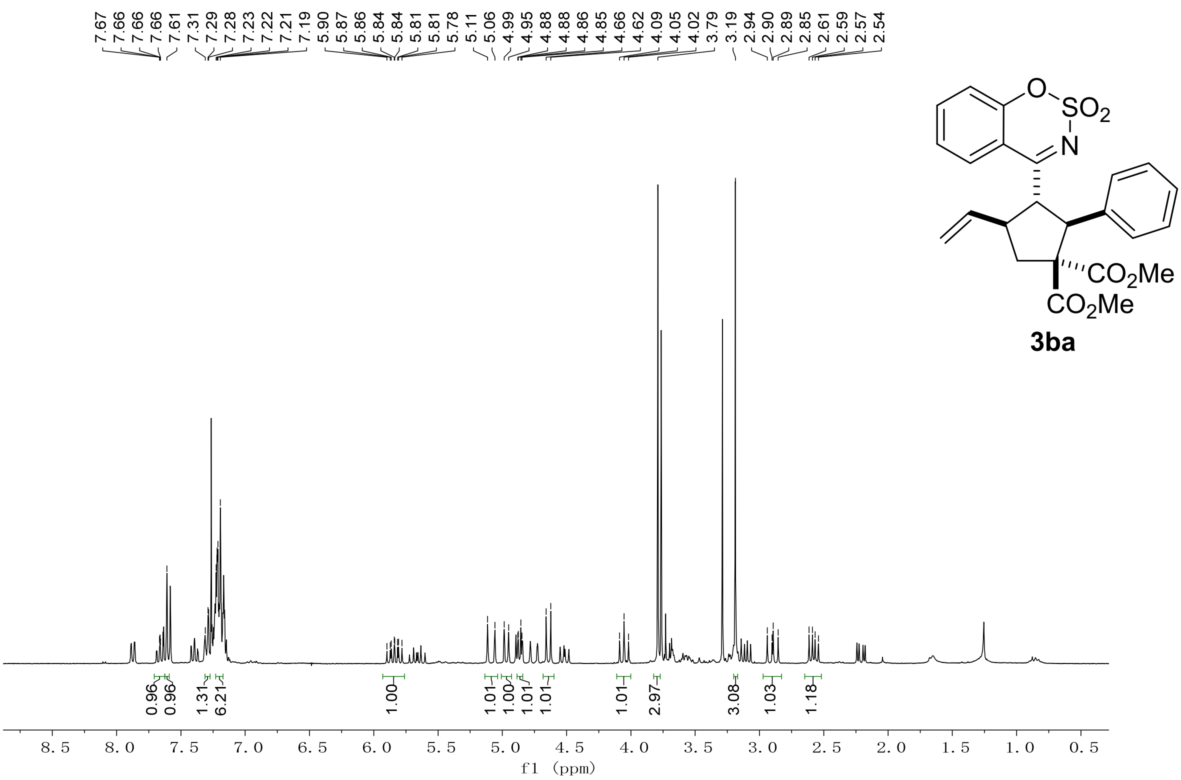
7.67 7.66 7.66 7.66 7.61 7.31 7.29 7.28 7.23 7.22 7.21 7.19 5.90 5.87 5.86 5.84 5.84 5.81 5.81 5.78 5.11 5.06 4.99 4.95 4.88 4.88 4.86 4.85 4.66 4.62 4.09 4.05 4.02 3.79 3.19 2.94 2.90 2.89 2.85 2.61 2.59 2.57 2.54
0.96 0.96 1.31 6.21 1.00 1.01 1.00 1.01 1.01 1.01 2.97 3.08 1.03 1.18
8.5 8.0 7.5 7.0 6.5 6.0 5.5 5.0 4.5 4.0 3.5 3.0 2.5 2.0 1.5 1.0 0.5
f1 (ppm)
O
SO2
N
CO2Me
CO2Me

3ba

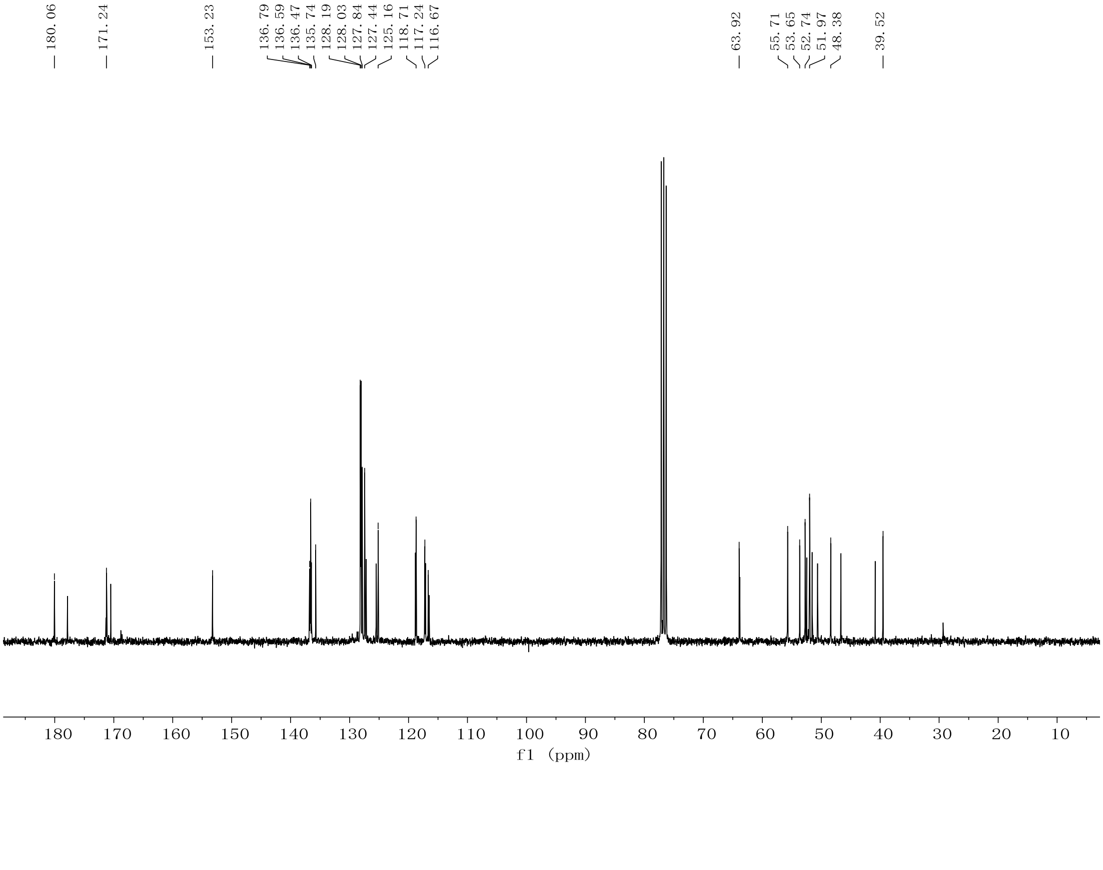
180.06 171.24 153.23 136.79 136.59 136.47 135.74 128.19 128.03 127.84 127.44 125.16 118.71 117.24 116.67 63.92 55.71 53.65 52.74 51.97 48.38 39.52
180 170 160 150 140 130 120 110 100 90 80 70 60 50 40 30 20 10
f1 (ppm)

X-Ray Crystallographic Data of 3-(2,2-dioxidobenzo[*e*][1,2,3]oxathiazin-4-yl)-2-phenyl-4-vinylcyclopentane-1,1-dicarbonitrile (3aa and 3aa’).

Crystallographic data for **3aa** and **3aa’** have been deposited with the Cam-bridge Crystallographic Data Centre as deposition number CCDC 1849601 and 1849602. These data can be obtained free of charge via www.ccdc.cam. ac.uk/data_request/cif, or by emailing data_request@ccdc.cam.ac.uk, or by contacting The Cambridge Crystallographic Data Centre, 12, Union Road, Cambridge CB2 1EZ, UK; fax: +44 1223 336033.

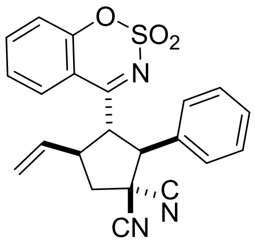

3aa

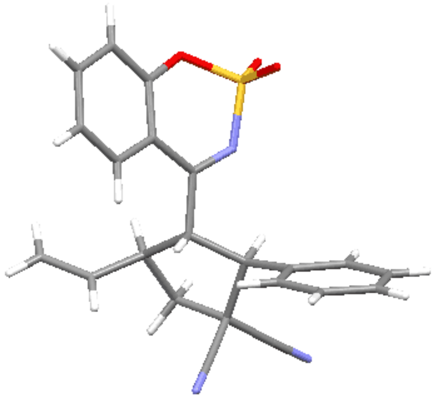

Table S1. Crystal data and structure refinement for **3aa**.

Identification code **3aa**

Empirical formula C22 H17 N3 O3 S

Formula weight 403.44

Temperature 293(2) K

Wavelength 0.71073 Å

Crystal system Monoclinic

Space group P 1 21/n 1

Unit cell dimensions a = 7.6209(15) Å a= 90°.

b = 12.073(2) Å b= 96.86(3)°.

c = 21.125(4) Å g = 90°.

Volume 1929.8(7) Å3

Z 4

Density (calculated) 1.389 Mg/m3

Absorption coefficient 0.197 mm-1

F(000) 840

Theta range for data collection 1.942 to 27.506°.

Index ranges -9<=h<=9, -15<=k<=15, -27<=l<=27
Reflections collected 14264
Independent reflections 4390 [R(int) = 0.0568]
Completeness to theta = 25.242° 99.4 %
Absorption correction None
Refinement method Full-matrix least-squares on F2
Data / restraints / parameters 4390 / 0 / 262
Goodness-of-fit on F2 1.225
Final R indices [I>2sigma(I)] R1 = 0.0732, wR2 = 0.1259
R indices (all data) R1 = 0.0846, wR2 = 0.1307
Extinction coefficientn/a
Largest diff. peak and hole 0.269 and -0.332 e.Å-3

Table S2. Atomic coordinates (x 10^4) and equivalent isotropic displacement parameters ($Å^2$ x 10^3) for **3aa**. U(eq) is defined as one third of the trace of the orthogonalized Uij tensor.

	x	y	z	U(eq)
S1	7351(1)	5078(1)	4472(1)	27(1)
O1	7202(2)	4450(2)	5127(1)	31(1)
O2	6892(3)	6189(2)	4596(1)	40(1)
O3	9006(2)	4842(2)	4264(1)	37(1)
N1	5803(3)	4529(2)	3971(1)	25(1)
N2	1001(3)	5104(2)	1761(1)	32(1)
N3	1511(3)	1595(2)	2117(1)	35(1)
C1	5367(3)	3503(2)	4029(1)	21(1)
C2	6124(3)	2798(2)	4560(1)	24(1)
C3	7070(3)	3297(2)	5094(1)	29(1)
C4	7829(4)	2703(3)	5611(1)	39(1)
C5	7656(4)	1565(3)	5598(1)	45(1)

C6	6733(4)	1031(3)	5079(1)	41(1)
C7	5966(4)	1645(2)	4566(1)	31(1)
C8	3958(3)	3066(2)	3529(1)	21(1)
C9	3865(3)	3739(2)	2910(1)	22(1)
C10	1902(3)	3579(2)	2615(1)	22(1)
C11	896(4)	3705(2)	3205(1)	28(1)
C12	2067(3)	3132(2)	3761(1)	25(1)
C13	1375(4)	2021(2)	3923(1)	31(1)
C14	925(4)	1744(3)	4480(2)	41(1)
C15	1376(3)	4423(2)	2124(1)	22(1)
C16	1647(3)	2467(2)	2325(1)	23(1)
C17	5211(3)	3505(2)	2458(1)	23(1)
C18	5835(4)	2442(2)	2351(1)	29(1)
C19	6962(4)	2266(3)	1895(1)	38(1)
C20	7483(4)	3134(3)	1538(1)	42(1)
C21	6903(4)	4197(3)	1646(1)	39(1)
C22	5784(3)	4379(2)	2105(1)	29(1)

Table S3. Bond lengths [Å] and angles [°] for **3aa**.

S1-O1	1.5935(19)
S1-O2	1.418(2)
S1-O3	1.413(2)
S1-N1	1.629(2)
O1-C3	1.397(3)
N1-C1	1.293(3)
N2-C15	1.137(3)

N3-C16	1.141(3)
C1-C2	1.470(3)
C1-C8	1.508(3)
C2-C3	1.399(3)
C2-C7	1.398(4)
C3-C4	1.377(4)
C4-H4	0.9300
C4-C5	1.380(5)
C5-H5	0.9300
C5-C6	1.388(4)
C6-H6	0.9300
C6-C7	1.384(4)
C7-H7	0.9300
C8-H8	0.9800
C8-C9	1.534(3)
C8-C12	1.579(3)
C9-H9	0.9800
C9-C10	1.562(3)
C9-C17	1.509(3)
C10-C11	1.547(3)
C10-C15	1.475(3)
C10-C16	1.479(3)
C11-H11A	0.9700
C11-H11B	0.9700
C11-C12	1.550(3)
C12-H12	0.9800
C12-C13	1.496(4)
C13-H13	0.9300

C13-C14	1.308(4)
C14-H14A	0.9300
C14-H14B	0.9300
C17-C18	1.397(4)
C17-C22	1.393(4)
C18-H18	0.9300
C18-C19	1.381(4)
C19-H19	0.9300
C19-C20	1.377(4)
C20-H20	0.9300
C20-C21	1.385(5)
C21-H21	0.9300
C21-C22	1.383(4)
C22-H22	0.9300

O1-S1-N1	104.42(11)
O2-S1-O1	104.24(12)
O2-S1-N1	109.16(12)
O3-S1-O1	109.47(12)
O3-S1-O2	119.84(13)
O3-S1-N1	108.61(11)
C3-O1-S1	116.25(16)
C1-N1-S1	120.22(18)
N1-C1-C2	123.0(2)
N1-C1-C8	116.0(2)
C2-C1-C8	120.9(2)
C3-C2-C1	118.9(2)
C7-C2-C1	123.8(2)

C7-C2-C3	117.3(2)
O1-C3-C2	119.8(2)
C4-C3-O1	117.3(2)
C4-C3-C2	122.9(3)
C3-C4-H4	120.9
C3-C4-C5	118.2(3)
C5-C4-H4	120.9
C4-C5-H5	119.4
C4-C5-C6	121.2(3)
C6-C5-H5	119.4
C5-C6-H6	120.1
C7-C6-C5	119.7(3)
C7-C6-H6	120.1
C2-C7-H7	119.6
C6-C7-C2	120.8(3)
C6-C7-H7	119.6
C1-C8-H8	109.0
C1-C8-C9	111.6(2)
C1-C8-C12	111.45(19)
C9-C8-H8	109.0
C9-C8-C12	106.63(19)
C12-C8-H8	109.0
C8-C9-H9	106.9
C8-C9-C10	102.67(19)
C10-C9-H9	106.9
C17-C9-C8	118.3(2)
C17-C9-H9	106.9
C17-C9-C10	114.52(19)

C11-C10-C9 102.09(19)

C15-C10-C9 111.2(2)

C15-C10-C11 112.3(2)

C15-C10-C16 109.0(2)

C16-C10-C9 110.5(2)

C16-C10-C11 111.7(2)

C10-C11-H11A 110.6

C10-C11-H11B 110.6

C10-C11-C12 105.7(2)

H11A-C11-H11B 108.7

C12-C11-H11A 110.6

C12-C11-H11B 110.6

C8-C12-H12 108.9

C11-C12-C8 104.61(19)

C11-C12-H12 108.9

C13-C12-C8 112.7(2)

C13-C12-C11 112.7(2)

C13-C12-H12 108.9

C12-C13-H13 117.5

C14-C13-C12 124.9(3)

C14-C13-H13 117.5

C13-C14-H14A 120.0

C13-C14-H14B 120.0

H14A-C14-H14B 120.0

N2-C15-C10 177.4(3)

N3-C16-C10 177.2(3)

C18-C17-C9 123.1(2)

C22-C17-C9 118.5(2)

C22-C17-C18	118.3(2)
C17-C18-H18	119.8
C19-C18-C17	120.4(3)
C19-C18-H18	119.8
C18-C19-H19	119.7
C20-C19-C18	120.6(3)
C20-C19-H19	119.7
C19-C20-H20	120.1
C19-C20-C21	119.8(3)
C21-C20-H20	120.1
C20-C21-H21	120.1
C22-C21-C20	119.9(3)
C22-C21-H21	120.1
C17-C22-H22	119.5
C21-C22-C17	121.0(3)
C21-C22-H22	119.5

Symmetry transformations used to generate equivalent atoms:

Table S4. Anisotropic displacement parameters ($Å^2$ x 10^3) for **3aa**. The anisotropic displacement factor exponent takes the form: -2p2[h2 a*2U11 + ...   + 2 h k a* b* U12]

	U11	U22	U33	U23	U13	U12
S1	28(1)	30(1)	24(1)	-7(1)	3(1)	-7(1)
O1	32(1)	39(1)	22(1)	-8(1)	1(1)	-7(1)

O2	47(1)	29(1)	44(1)	-14(1)	3(1)	-7(1)
O3	30(1)	51(1)	30(1)	-10(1)	7(1)	-10(1)
N1	26(1)	24(1)	24(1)	-3(1)	-2(1)	-1(1)
N2	34(1)	25(1)	34(1)	5(1)	-5(1)	0(1)
N3	41(1)	26(1)	36(1)	-2(1)	-2(1)	-2(1)
C1	22(1)	23(1)	18(1)	-3(1)	3(1)	1(1)
C2	21(1)	31(1)	20(1)	3(1)	0(1)	0(1)
C3	25(1)	39(2)	24(1)	1(1)	3(1)	-2(1)
C4	32(2)	62(2)	23(1)	5(1)	-4(1)	-3(2)
C5	34(2)	64(2)	34(2)	25(2)	-5(1)	0(2)
C6	36(2)	39(2)	45(2)	19(1)	-4(1)	-4(1)
C7	28(1)	32(2)	32(1)	6(1)	-3(1)	0(1)
C8	23(1)	18(1)	20(1)	-1(1)	-1(1)	0(1)
C9	25(1)	18(1)	20(1)	-1(1)	-2(1)	-1(1)
C10	22(1)	19(1)	22(1)	0(1)	-1(1)	1(1)
C11	28(1)	30(1)	26(1)	0(1)	4(1)	4(1)
C12	28(1)	24(1)	22(1)	-1(1)	3(1)	0(1)
C13	31(2)	28(1)	33(1)	-2(1)	2(1)	-2(1)
C14	47(2)	33(2)	45(2)	6(1)	12(2)	-7(2)
C15	23(1)	18(1)	25(1)	-3(1)	-2(1)	0(1)
C16	22(1)	24(1)	22(1)	4(1)	-2(1)	1(1)
C17	23(1)	26(1)	19(1)	-2(1)	-3(1)	-3(1)
C18	29(1)	29(1)	30(1)	-3(1)	4(1)	0(1)
C19	31(2)	44(2)	39(2)	-13(1)	4(1)	4(1)
C20	29(2)	67(2)	32(2)	-6(2)	7(1)	-1(2)
C21	29(2)	54(2)	34(2)	11(1)	4(1)	-7(1)
C22	26(1)	30(2)	31(1)	4(1)	-1(1)	-1(1)

Table S5. Hydrogen coordinates (x 104) and isotropic displacement parameters (Å^2 x 10^3) for **3aa**.

	x	y	z	U(eq)
H4	8442	3058	5961	47
H5	8166	1149	5942	53
H6	6631	263	5077	49
H7	5337	1286	4221	37
H8	4222	2293	3437	25
H9	4000	4520	3033	26
H11A	-253	3351	3131	33
H11B	729	4481	3300	33
H12	2130	3608	4139	30
H13	1255	1488	3603	37
H14A	1026	2256	4811	49
H14B	504	1035	4545	49
H18	5489	1848	2587	35
H19	7374	1556	1829	46
H20	8222	3007	1226	51
H21	7264	4787	1409	47
H22	5409	5096	2179	35

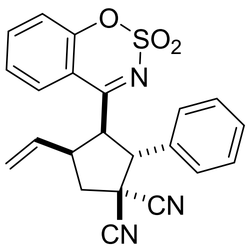

3aa'

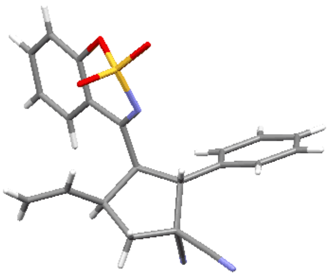

Table S6. Crystal data and structure refinement for **3aa'**.

Identification code **3aa'**

Empirical formula C22 H17 N3 O3 S

Formula weight 403.44

Temperature 293(2) K

Wavelength 0.71073 Å

Crystal system Triclinic

Space group P -1

Unit cell dimensions a = 8.2461(16) Å a= 104.17(3)°.

b = 10.300(2) Å b= 99.94(3)°.

c = 12.879(3) Å g = 105.52(3)°.

Volume 988.1(4) Å3

Z 2

Density (calculated) 1.356 Mg/m3

Absorption coefficient 0.193 mm-1

F(000) 420

Crystal size ? x ? x ? mm3

Theta range for data collection 1.687 to 27.470°.

Index ranges -10<=h<=10, -13<=k<=13, -16<=l<=16

Reflections collected 13604

Independent reflections 4495 [R(int) = 0.0431]

Completeness to theta = 25.242° 99.6 %

Absorption correction None

Refinement method Full-matrix least-squares on F2

Data / restraints / parameters 4495 / 12 / 262

Goodness-of-fit on F2 1.101

Final R indices [I>2sigma(I)] R1 = 0.0567, wR2 = 0.1206

R indices (all data) R1 = 0.0642, wR2 = 0.1254

Extinction coefficientn/a

Largest diff. peak and hole 0.430 and -0.350 e.Å-3

Table S7. Atomic coordinates (x 104) and equivalent isotropic displacement parameters (Å^2 x 10^3) for **3aa'**. U(eq) is defined as one third of the trace of the orthogonalized Uij tensor.

	x	y	z	U(eq)
S1	1766(1)	3918(1)	4279(1)	28(1)
O1	3526(2)	3614(2)	4112(1)	31(1)
O2	1651(2)	4944(2)	3736(1)	34(1)
O3	398(2)	2605(2)	3940(1)	38(1)
N1	3973(3)	6073(2)	10584(2)	43(1)
N2	-335(3)	7221(2)	9281(2)	45(1)
N3	2119(2)	4611(2)	5611(1)	26(1)
C1	4257(3)	2981(2)	4831(2)	26(1)
C2	5259(3)	2191(2)	4445(2)	32(1)
C3	6106(3)	1639(2)	5159(2)	34(1)
C4	5932(3)	1862(2)	6237(2)	31(1)
C5	4932(3)	2661(2)	6610(2)	28(1)
C6	4062(2)	3252(2)	5914(2)	24(1)
C7	3060(2)	4170(2)	6275(2)	23(1)
C8	3024(3)	4590(2)	7480(2)	24(1)
C9	1696(3)	3360(2)	7731(2)	34(1)
C10	513(3)	4076(2)	8291(2)	31(1)
C11	1604(3)	5652(2)	8782(2)	26(1)
C12	2483(2)	5920(2)	7836(2)	23(1)
C13	635(3)	2091(3)	6774(2)	48(1)

C14	383(4)	782(3)	6761(4)	82(1)
C15	3860(2)	7358(2)	8154(2)	24(1)
C16	3314(3)	8514(2)	8089(2)	28(1)
C17	4508(3)	9862(2)	8392(2)	35(1)
C18	6259(3)	10076(2)	8752(2)	38(1)
C19	6822(3)	8945(3)	8821(2)	41(1)
C20	5627(3)	7595(2)	8530(2)	33(1)
C21	2944(3)	5903(2)	9809(2)	30(1)
C22	527(3)	6555(2)	9055(2)	31(1)

Table S8. Bond lengths [Å] and angles [°] for **3aa’**.

S1-O1	1.6028(16)
S1-O2	1.4172(15)
S1-O3	1.4195(17)
S1-N3	1.6288(18)
O1-C1	1.400(2)
N1-C21	1.136(3)
N2-C22	1.139(3)
N3-C7	1.300(2)
C1-C2	1.377(3)
C1-C6	1.399(3)
C2-H2	0.9300
C2-C3	1.380(3)

C3-H3	0.9300
C3-C4	1.390(3)
C4-H4	0.9300
C4-C5	1.376(3)
C5-H5	0.9300
C5-C6	1.402(3)
C6-C7	1.460(3)
C7-C8	1.513(3)
C8-H8	0.9800
C8-C9	1.578(3)
C8-C12	1.541(3)
C9-H9	0.9800
C9-C10	1.544(3)
C9-C13	1.490(3)
C10-H10A	0.9700
C10-H10B	0.9700
C10-C11	1.537(3)
C11-C12	1.565(3)
C11-C21	1.487(3)
C11-C22	1.472(3)
C12-H12	0.9800
C12-C15	1.515(3)
C13-H13	0.9300
C13-C14	1.304(4)
C14-H14A	0.9300

C14-H14B	0.9300
C15-C16	1.397(3)
C15-C20	1.387(3)
C16-H16	0.9300
C16-C17	1.386(3)
C17-H17	0.9300
C17-C18	1.378(3)
C18-H18	0.9300
C18-C19	1.380(3)
C19-H19	0.9300
C19-C20	1.389(3)
C20-H20	0.9300

O1-S1-N3	104.95(9)
O2-S1-O1	104.33(9)
O2-S1-O3	119.56(9)
O2-S1-N3	110.16(9)
O3-S1-O1	108.63(9)
O3-S1-N3	108.22(10)
C1-O1-S1	116.74(13)
C7-N3-S1	118.94(14)
C2-C1-O1	116.76(18)
C2-C1-C6	123.05(18)
C6-C1-O1	120.00(17)
C1-C2-H2	120.8

C1-C2-C3	118.4(2)
C3-C2-H2	120.8
C2-C3-H3	119.7
C2-C3-C4	120.6(2)
C4-C3-H3	119.7
C3-C4-H4	120.0
C5-C4-C3	120.09(19)
C5-C4-H4	120.0
C4-C5-H5	119.5
C4-C5-C6	121.06(19)
C6-C5-H5	119.5
C1-C6-C5	116.74(18)
C1-C6-C7	119.48(17)
C5-C6-C7	123.71(18)
N3-C7-C6	123.83(17)
N3-C7-C8	117.02(17)
C6-C7-C8	119.08(16)
C7-C8-H8	108.5
C7-C8-C9	110.71(16)
C7-C8-C12	113.58(16)
C9-C8-H8	108.5
C12-C8-H8	108.5
C12-C8-C9	106.82(16)
C8-C9-H9	108.1
C10-C9-C8	104.45(16)

C10-C9-H9 108.1

C13-C9-C8 117.19(19)

C13-C9-H9 108.1

C13-C9-C10 110.66(19)

C9-C10-H10A 110.7

C9-C10-H10B 110.7

H10A-C10-H10B 108.8

C11-C10-C9 105.02(17)

C11-C10-H10A 110.7

C11-C10-H10B 110.7

C10-C11-C12 102.00(16)

C21-C11-C10 110.94(17)

C21-C11-C12 110.52(16)

C22-C11-C10 112.18(17)

C22-C11-C12 113.20(17)

C22-C11-C21 107.98(17)

C8-C12-C11 102.23(15)

C8-C12-H12 107.2

C11-C12-H12 107.2

C15-C12-C8 118.37(16)

C15-C12-C11 114.00(16)

C15-C12-H12 107.2

C9-C13-H13 117.4

C14-C13-C9 125.1(3)

C14-C13-H13 117.4

C13-C14-H14A 120.0

C13-C14-H14B 120.0

H14A-C14-H14B 120.0

C16-C15-C12 118.01(17)

C20-C15-C12 123.67(18)

C20-C15-C16 118.30(19)

C15-C16-H16 119.6

C17-C16-C15 120.75(19)

C17-C16-H16 119.6

C16-C17-H17 119.9

C18-C17-C16 120.2(2)

C18-C17-H17 119.9

C17-C18-H18 120.1

C17-C18-C19 119.8(2)

C19-C18-H18 120.1

C18-C19-H19 119.9

C18-C19-C20 120.2(2)

C20-C19-H19 119.9

C15-C20-C19 120.8(2)

C15-C20-H20 119.6

C19-C20-H20 119.6

N1-C21-C11178.7(2)

N2-C22-C11177.7(2)

Symmetry transformations used to generate equivalent atoms:

Table S9. Anisotropic displacement parameters (Å^2 x 10^3) for **3aa'**. The anisotropic displacement factor exponent takes the form: -2p2[h2 a*2U11 + ... + 2 h k a* b* U12]

	U11	U22	U33	U23	U13	U12
S1	30(1)	28(1)	20(1)	5(1)	-1(1)	7(1)
O1	39(1)	36(1)	20(1)	9(1)	6(1)	15(1)
O2	39(1)	36(1)	24(1)	13(1)	0(1)	12(1)
O3	38(1)	31(1)	31(1)	4(1)	-5(1)	0(1)
N1	51(1)	54(1)	25(1)	10(1)	3(1)	24(1)
N2	40(1)	57(1)	45(1)	15(1)	14(1)	25(1)
N3	28(1)	26(1)	20(1)	6(1)	2(1)	7(1)
C1	31(1)	24(1)	20(1)	5(1)	1(1)	7(1)
C2	39(1)	29(1)	25(1)	4(1)	10(1)	10(1)
C3	35(1)	28(1)	37(1)	5(1)	9(1)	12(1)
C4	35(1)	27(1)	32(1)	10(1)	4(1)	12(1)
C5	32(1)	26(1)	22(1)	6(1)	4(1)	9(1)
C6	27(1)	22(1)	20(1)	5(1)	3(1)	7(1)
C7	24(1)	20(1)	19(1)	4(1)	1(1)	3(1)
C8	28(1)	24(1)	20(1)	6(1)	3(1)	10(1)
C9	40(1)	30(1)	34(1)	14(1)	11(1)	10(1)
C10	32(1)	32(1)	27(1)	12(1)	7(1)	5(1)
C11	27(1)	31(1)	20(1)	8(1)	4(1)	11(1)
C12	25(1)	25(1)	17(1)	7(1)	3(1)	8(1)
C13	43(1)	35(1)	57(2)	4(1)	22(1)	2(1)

C14	59(2)	31(2)	140(4)	8(2)	22(2)	7(1)
C15	27(1)	25(1)	17(1)	4(1)	4(1)	8(1)
C16	31(1)	28(1)	25(1)	6(1)	4(1)	12(1)
C17	44(1)	28(1)	35(1)	9(1)	9(1)	14(1)
C18	39(1)	26(1)	40(1)	6(1)	3(1)	2(1)
C19	27(1)	39(1)	49(1)	11(1)	0(1)	6(1)
C20	30(1)	30(1)	36(1)	11(1)	3(1)	11(1)
C21	38(1)	34(1)	20(1)	8(1)	8(1)	16(1)
C22	30(1)	38(1)	25(1)	11(1)	7(1)	11(1)

Table S10. Hydrogen coordinates (x 10^4) and isotropic displacement parameters (Å^2 x 10^3) for **3aa’**.

	x	y	z	U(eq)
H2	5363	2034	3721	38
H3	6801	1112	4916	41
H4	6493	1471	6707	38
H5	4830	2810	7334	33
H8	4189	4772	7938	29
H9	2350	3046	8275	41
H10A	-544	3947	7753	38
H10B	200	3684	8868	38

H12	1567	5872	7218	27
H13	110	2245	6136	58
H14A	886	584	7383	98
H14B	-299	45	6129	98
H16	2135	8377	7839	34
H17	4126	10624	8352	42
H18	7060	10980	8948	46
H19	8004	9088	9063	49
H20	6016	6842	8588	39
